# Effects of aquatic exercise on improving body composition and muscle strength in the older adults: a systematic review and meta-analysis of randomized controlled trials

**DOI:** 10.3389/fpubh.2025.1726568

**Published:** 2026-02-10

**Authors:** Yuan Gao, Wenze Deng, Qiancheng Zeng, Yichen Liu, Xiaofu Tang, Sitian Fang, Liang Hao, Hongbo Li

**Affiliations:** 1 Nanchang County People’s Hospital, Nanchang, Jiangxi, China; 2 Department of Orthopedics, The Second Affiliated Hospital, Jiangxi Medical College, Nanchang University, Nanchang, Jiangxi, China; 3 School of Nursing, Jiangxi Medical College, Nanchang University, Nanchang, Jiangxi, China; 4 Huankui Academy, Jiangxi Medical College, Nanchang University, Nanchang, Jiangxi, China; 5 Department of Clinical Medicine, The First Clinical College of Nanchang University, Nanchang, Jiangxi, China; 6 The Second Affiliated Hospital, Jiangxi Medical College, Nanchang University, Nanchang, China; 7 Institute of Orthopedics of Jiangxi Province, Nanchang, Jiangxi, China; 8 Jiangxi Provincial Key Laboratory of Spine and Spinal Cord Disease, Nanchang, Jiangxi, China; 9 Institute of Minimally Invasive Orthopedics, Nanchang University, Nanchang, Jiangxi, China

**Keywords:** aging, aquatic exercise, body composition, meta-analysis, muscle strength, systematic review

## Abstract

**Background:**

Due to the challenges posed by aging such as decreased physical abilities and higher susceptibility to chronic illnesses, effective exercise interventions are crucial for older individuals. Despite the potential benefits of aquatic exercise, there is a lack of robust evidence supporting their efficacy. To address this gap, a systematic review and meta-analysis were conducted in this study to comprehensively assess the impact of aquatic exercise on muscle strength, body composition, and related physical (flexibility, mobility) and metabolic indicators (lipid profiles) in the older adults, aiming to inform the development of intervention strategies.

**Methods:**

We searched seven databases (ClinicalTrials.gov, Scopus, Medline, PubMed, Embase, Web of Science, and the Cochrane Library) from inception to August 1, 2025. Our analysis included 19 Randomized controlled trials (RCTs) (*n* = 866 participants) and adhered to Preferred Reporting Project for Systematic Reviews and Meta-analyses (PRISMA) guidelines, employing meta-analytical methods for outcome assessment. Meta-analyses and meta-regressions were conducted to determine the mean difference Additionally, heterogeneity, risk of bias, and certainty of evidence were evaluated.

**Results:**

The meta-analysis findings indicated significant enhancements in muscle strength and flexibility among older adults engaging in aquatic exercise. Additionally, improvements in functional mobility were observed, as evidenced by better performance on the Timed Up and Go test. Moreover, reductions in body fat percentage and total cholesterol levels were observed as notable improvements in metabolic indicators. Nonetheless, the intervention did not yield significant effects on body weight, body mass index, or levels of high-density lipoprotein cholesterol, low-density lipoprotein cholesterol, and triglycerides.

**Conclusion:**

This review indicates that aquatic exercise may serve as a viable intervention strategy for preserving muscle function, flexibility, and lipid homeostasis in the older adults population, thereby offering a novel perspective on maintaining functional independence. These findings underscore the potential application of aquatic exercise in geriatric care; however, a multicenter study with large sample sizes and long-term follow-up is necessary to further validate the generalizability of the metabolic benefits and long-term safety.

**Systematic review registration:**

Identifier CRD42024568443.

## Highlights

- Aquatic exercise significantly improved muscle strength and flexibility.
- Aquatic exercise reduced TUG test time, indicating enhanced functional capacity.
- Lipid and cholesterol levels in aquatic exercise were significantly reduced.

## 1 Introduction

Aging is essentially characterized by progressive multi-system degeneration of the body, accompanied by a decline in physical fitness and an exponential increase in the risk of chronic diseases (1). The core manifestation of this is a significant decline in cardiorespiratory endurance and muscle strength (2, 3). The former directly restricts daily activities like stair climbing and walking, while the latter significantly contributes to the decline of functional independence and is closely linked to chronic diseases such as diabetes and atherosclerosis (4–6). Simultaneously, aging frequently coincides with weight gain, augmented total body fat, and the buildup of abdominal fat (7, 8), establishing a detrimental cycle of “metabolic disorders - functional decline”: the enlargement of adipose tissue discharges inflammatory factors, hindering muscle protein synthesis. Concurrently, muscle loss diminishes the basal metabolic rate, consequently intensifying fat accumulation (9–11). These alterations ultimately elevate the susceptibility to obesity, cardiovascular disease, and diabetes, while also resulting in constraints in daily activities, heightened fall risks, and increased mortality (12, 13). Furthermore, while the deterioration decline in muscle strength and alterations in body composition leads contribute to limited daily activities, decreased balance, and increased a heightened risk of falls and disability (14–16).

To effectively counteract age-related physiological decline and extend active life expectancy, international guidelines recommend that exercise interventions be specifically tailored to individual goals and personalized according to exercise mode, frequency, duration, and intensity (17). Furthermore, multimodal exercise programs are advocated, incorporating aerobic activity, progressive resistance training, balance exercises, and mobility training delivered through both structured sessions and lifestyle-integrated activities (17). Within such comprehensive frameworks, aerobic exercise enhances cardiorespiratory and metabolic parameters, while resistance training boosts muscle strength, balance, and diminishes fall risk (18, 19). Nonetheless, executing these core activities on land presents several challenges. For instance, for older adults with conditions like osteoarthritis, participation in land-based exercise can be hindered by significant barriers, including exercise-induced pain, physical limitations, and environmental concerns such as unsafe walking surfaces (20). Additionally, accurately controlling the intensity of land-based aerobic exercise is difficult, potentially causing fatigue or injuries in older adults individuals due to over-exertion (21).

Conversely, aquatic exercise leverages the unique physical properties of water to promote mobility and muscle strengthening (22). The buoyancy of water counteracts gravity, significantly reducing weight-bearing loads and alleviating pain, thereby offering a safer, low-impact alternative to land-based training (23). Simultaneously, water provides omnidirectional resistance that intensifies with movement speed, necessitating greater muscle activation and stabilizing effort for effective strengthening (23). The water’s temperature aids in muscle relaxation and alleviates post-exercise soreness (24). Additionally, activities such as “water walking” and “water Tai Chi” combine enjoyment and safety, thereby enhancing exercise adherence among older adults (25). Early studies have indicated that water exercise can positively affect weight and blood lipid levels in patients with chronic diseases (26–28). Recent systematic reviews have further demonstrated its efficacy in improving muscle strength and physical function in older adults (29, 30).

Despite the potential benefits of aquatic exercise, the current evidence has limitations. Most studies tend to focus on individual outcomes rather than providing a comprehensive evaluation of “strength-body composition-function.” Additionally, there is a scarcity of research involving healthy older adults, particularly in elucidating the preventive impact on individuals without existing diseases. The objective of this study is to address existing gaps by conducting a systematic review and meta-analysis to assess the impact of aquatic exercise on muscle strength, body composition, and related physical (flexibility, mobility) and metabolic indicators (lipid profiles) in healthy older adults (31–33). We hypothesized that, due to the unique resistive and buoyant properties of water, aquatic exercise would elicit significant improvements in muscle strength and physical function, as well as favorable changes in body composition and lipid profiles compared to inactive controls. These findings are expected to provide evidence-based guidance for optimizing geriatric exercise recommendations, facilitating the translation of “healthy aging” principles into practical applications.

## 2 Methods

This review as conducted in accordance with the Preferred Reporting Project for Systematic Reviews and Meta-analyses (PRISMA) statement (34) and was registered in PROSPERO (CRD42024568443).

### 2.1 Search strategy

To identify all potentially eligible studies, two independent researchers conducted searches in the ClinicalTrial.gov, Scopus, Medline, PubMed, EMBASE, Web of Science, and Cochrane Library databases. Randomized controlled trials (RCTs) investigating the effects of aquatic exercise on body composition and muscle strength in older adults were retrieved from inception until August 2025. The search strategy utilized specific keywords combined with Boolean operators (AND, OR) to refine the results. The complete search strategy for each database is detailed in the Supplementary Table S1.

### 2.2 Selection criteria

The inclusion and exclusion criteria were established based on the PICOS (Population, Intervention, Comparison, Outcomes, and Study design) framework. Articles were included if they met the following criteria: (1) Study design: The included studies were randomized controlled trials (RCTs); (2) Population: The study population consisted of healthy older adults, aged 60 years and above, following the World Health Organization classification (35, 36). However, some studies included Aboriginal and Torres Strait Islander peoples. Given known differences in life expectancy and health trajectories in this group, the minimum age for the ‘older adults’ subgroup among Indigenous Australians is set at 50 years, as defined by the Australian Institute of Health and Welfare (37, 38). (3) Interventions: The experimental group participated in aquatic exercise programs. There were no restrictions regarding the specific type, frequency, intensity, or duration of the aquatic intervention. (4) Comparisons: The control group participated in land-based exercise, maintained their usual daily activities (sedentary control), or received no exercise therapy. (5) Outcomes: The study reported at least one of the following outcomes: primary outcomes included muscle strength [e.g., grip strength, 30-s chair stand test (30CST), arm curl test] and body composition (e.g., body fat mass, lipid profiles such as HDL-C and LDL-C); secondary outcomes included flexibility (e.g., sit-and-reach, back scratch test), mobility (e.g., Timed Up and Go [TUG] test), and anthropometric measures (e.g., body weight, BMI, waist-to-hip ratio). Studies were excluded if they met the following criteria: (1) Non-English literature; (2) Literature from which data could not be extracted; (3) Other types of articles.

Furthermore, lipid metabolism markers, specifically HDL-C, LDL-C, although not conventionally considered body composition parameters, are frequently studied in conjunction with fat distribution. Studies have demonstrated their beneficial impact on body composition. By incorporating these markers into the analysis, a more thorough evaluation of the effects of aquatic exercise on population health and wellness can be achieved, encompassing both structural and metabolic facets of body composition.

### 2.3 Literature screening and data extraction

All retrieved articles were imported into EndNote for deduplication. Two reviewers (WZD and QCZ) screened articles by title and abstract, followed by a full evaluation based on predefined criteria. Disagreements were resolved by a third reviewer (YG). Prior to the formal screening, a calibration exercise was performed on a random sample of 10 studies to ensure consistency between the reviewers. Key information from each study (e.g., study design, participant characteristics, outcome measures) was independently extracted using standardized Excel sheets. Missing data prompted reviewers to contact corresponding authors for supplementation via email.

### 2.4 Bias assessment

Two reviewers independently assessed the risk of bias in the included studies by version 2 of the Cochrane risk-of-bias tool for randomized trials (ROB 2) (39) for RCTs to assess the methodological quality of the included studies.

### 2.5 Statistical analysis

A descriptive analysis was conducted to summarize the fundamental characteristics of the included studies. Given that the study encompassed measurement data, the mean difference (MD) was employed as the metric for effect size analysis. The point estimate and the corresponding 95% confidence interval (CI) were provided for each effect size. In addition, some of the included studies had multiple intervention groups. To avoid omissions and double counting, we combined them according to the requirements of the Cochrane Handbook (40).

Heterogeneity was considered significant when *p* < 0.1 or *I*^2^ > 50%. If substantial heterogeneity was detected, a random-effects model was used; otherwise, a fixed-effects model was used. Subgroup analyses were performed to explore sources of heterogeneity. In subgroup analyses, a subgroup difference test was performed. If *p* < 0.1 or *I^2^* > 50%, the stratified study characteristic was considered to contribute to identified heterogeneity. Sensitivity analysis was conducted to assess the robustness of the outcomes regarding significant disparities and Meta-regression analyses were also performed to further assess sources of heterogeneity. The meta-analysis used Review Manager (Rev Man), version 5.4, and meta-regression was performed with Stata, version 16.0.

## 3 Results

### 3.1 Study selection

**Figure 1:**
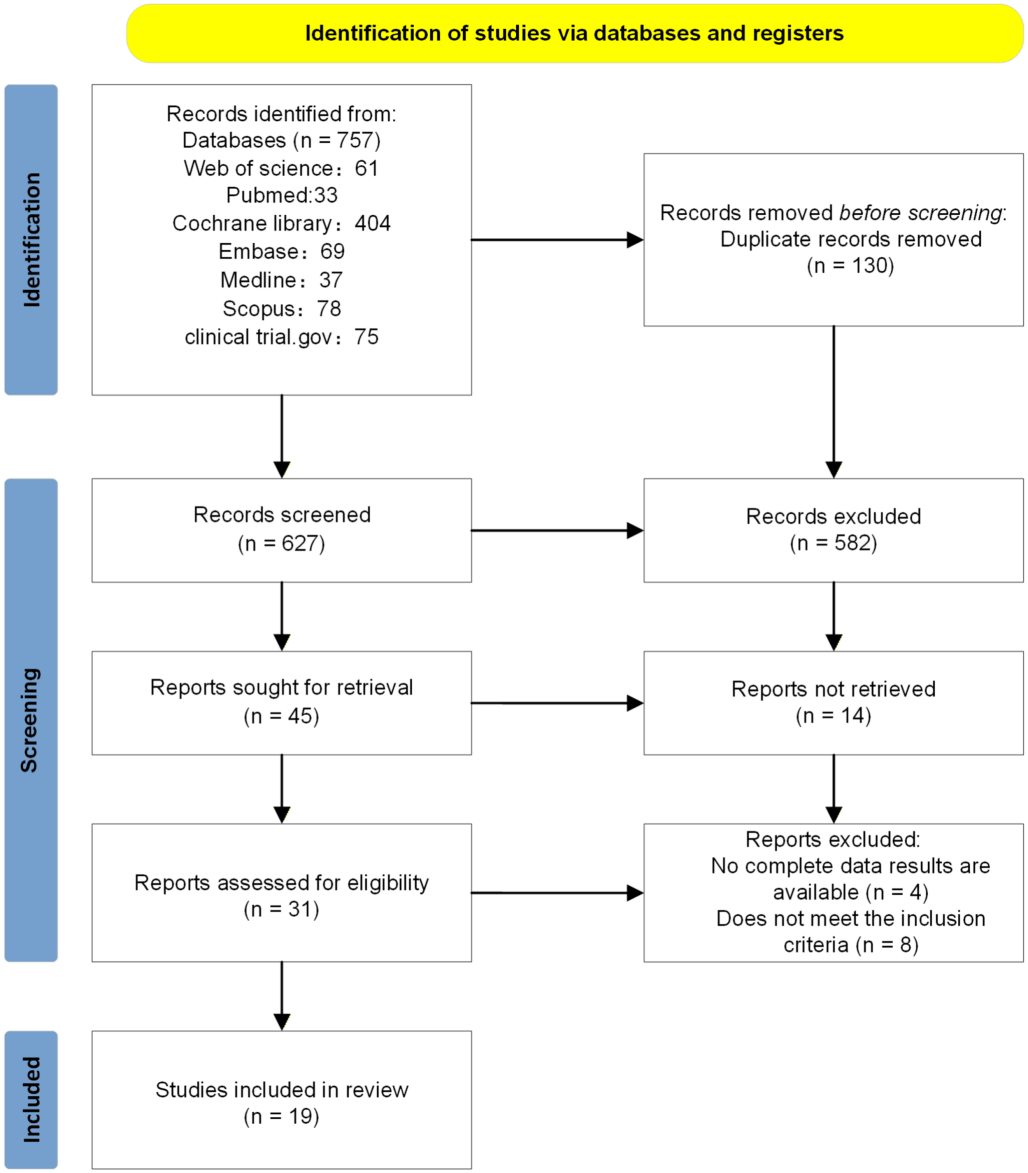
PRISMA flow diagram of the study selection process.

The systematic search initially retrieved 757 studies, of which 130 were duplicates. Titles and abstracts of 627 articles were screened, leading to 31 articles selected for full-text review. Ultimately, 12 studies were excluded, and 19 studies (41–59) were considered eligible for qualitative synthesis and quantitative analysis. The PRISMA flow chart of the review process is shown in Figure 1.

### 3.2 Study characteristics

We systematically collected core characteristics of each study, covering details such as author, publication year, country of origin, study design, intervention, number of participants, study duration, age range, gender distribution, and the outcomes measured (Table 1). The quality assessment results for all included studies are presented in Supplementary Figure S1. Overall, seven studies (42–44, 47, 49, 52, 54) demonstrated a low risk of bias and good applicability across all domains; however, the remaining studies exhibited a risk of bias in at least one domain. The summarized results of the meta-analysis are provided in Table 2.

**Table 1:** Basic characteristics of the included studies.

Id	Author/Year	Nation	Study design	Intervention	N(T/C)	Duration	Age (mean ± SD)	Man	Woman	Outcome
							T/C	T/C	T/C	
1	Cox et al (2010) (44)	Australian	RCT	Swimming	44/42	6 m/12 m	55.8 (4.5)/55.2 (4.8)	0/0	44/42	⑦⑧⑨⑩⑪⑫⑬⑭⑮⑯⑰
2	Ruoti et al. (1994) (53)	Panama	RCT	Non-swimming water exercise	12/8	12w	65 (5.29)/56 (6.78)	2/3	10/5	⑲
3	Bergamin et al. (2013) (57)	Italy	RCT	Water-based exercise	T:17	24w	NP	NP	NP	①⑦⑧⑱⑲
					C1:17					
					C2:19					
4	Kang et al. (51) (2024)	Korea	RCT	Water-based exercise	T:10	16w	T:68.34 (1.64)	T:0	10	⑦⑱⑲
					C1:10		C1:67.71 (1.35)	C1:0	C1:10	
					C2:10		C2:68.59 (1.83)	C2:0	C2:10	
5	Martínez-Rodríguez et al. (2021)(47)	Spain	RCT	Water resistance exercise	17/17	14w	69.6 (5.0)/67.7 (3.6)	0/0	17/17	⑦⑪⑱⑲
6	Kieffer et al. (2012) (49)	America	RCT	Water-based exercise	11/15	8w	75.6 (4.8)/79.6 (10.1)	4/7	11/4	②
7	Naylor et al. (2020) (56)	Australian	RCT	Water walking	T:20	24w/48w	T:62.6 (6.7)	T:0	20	⑦⑧⑨⑩⑪⑱⑲
					C1:20		C1:62.7 (7.0)	C1:0	C1:20	
					C2:20		C2:62.1 (7.0)	C2:0	C2:20	
8	Takeshima et al. (2002) (58)	Japan	RCT	Water-based exercise	15/15	12w	69.3 (4.5)/69.3 (3.3)	0/0	15/15	⑦⑫⑬⑭⑮⑯⑰
9	Oh et al. (2014) (45)	Korea	RCT	Water-based exercise	32/34	10w	74.71 (2.9)/68.21 (4.4)	NP	NP	④⑤⑥
10	Taunton et al. (1996) (48)	England	RCT	Water-based exercise	14/13	12w	70 (3.2)/70 (3.2)	0/0	14/13	①③⑧⑪
11	Bocalini et al. (2008) (59)	Brazil	RCT	Water-based exercise	T:27	12w	T:63 (1)	T:0	T:27	②③④⑤⑦
					C1:25		C1:64 (1)	C1:0	C1:25	
					C2:20		C2:64 (1)	C2:0	C2:20	
12	Ochoa-Martínez et al. (2019) (46)	Mexico	RCT	Water-based exercise	10/16	12w	67.5 (5.4)/67.4 (4.7)	0/0	16/10	⑦⑨⑮⑰
13	Martínez-Rodríguez et al. (2021) (47)	Spain	RCT	Water resistance exercise	17/17	14w	69.6 (5.01)/67.7 (3.60)	0/0	17/17	①⑨⑩⑪
14	Sanders et al. (2016) (41)	America	RCT	Water-based exercise	13/13	12w	70.8 (4.0)/70.1 (3.2)	0/0	13/13	②③④⑤
15	Askari et al. (2018) (43)	Iran	RCT	Water-based exercise	30/30	6w	69.9 (5.1)/68.0 (5.0)	30/30	0/0	⑥
16	Mary et al. (2013) (55)	America	RCT	Water-based exercise	43/17	16w	73.6 (13.5)/72.8 (27.4)	0/0	43/17	③④⑦
17	Tsourlou et al. (2006) (50)	Greece	RCT	Water resistance exercise	12/10	24w	69.3 (1.9)/68.4 (6.7)	0/0	12/10	④⑥⑦⑧⑱
18	Farinha et al. (2021) (54)	Portugal	RCT	Continuous aerobic water exercise	T1:25	28w	T1:71.44 (4.84)	T1:5	T1:20	①②③④⑤⑦⑧⑨⑫⑬⑲
				Intermittent aerobic water exercise	T2:28		T2:72.64 (5.22)	T2:3	T2:25	
				Combined water exercise	T3:29		T3:71.90 (5.67)	T3:7	T3:22	
					C:20		C:73.60 (5.25)	C:9	C:11	
19	Nosrani et al. (2024) (52)	Iran	RCT	Combined water exercise	19/13	28w	71.16 (5.91)/73.38 (5.81)	5/6	14/7	⑦⑧⑨⑫⑬⑭⑮⑯⑰⑲

N, number of participants; T, test group; C, control group; C1, land-based exercise; C2, no intervention; w, week; m, month; NP, not report; RCT, randomized controlled trial. ① Hand grip test; ② 30-s chair stand test (30CST); ③ Arm curl test; ④ Sit and reach; ⑤ Back scratch; ⑥ Timed Up and Go (TUG); ⑦ Body weight; ⑧ BMI; ⑨ Waist circumference; ⑩ Hip circumference; ⑪ Waist-to-hip ratio; ⑫ Arm circumference; ⑬ Thigh circumference; ⑭ Total Cholesterol; ⑮ High density lipoprotein cholesterol (HDL-C); ⑯ Low Density Lipoprotein (LDL-C); ⑰ Triglyceride; ⑱ Body fat mass; ⑲ Body Fat Percentage. The study conducted by Kay et al. (2010) included Aboriginal Australians, so the Age (mean ± SD) was 55.8(4.5)/55.2(4.8).

**Table 2:** Summary table of meta-analysis results.

Outcome indicator	Subgroup	*N*	Results of heterogeneity		Model of effect	Results of meta-analysis	
			*p* value	*I^2^*		Effect Size (95%CI)	*p* value
Primary outcome							
1. Muscle strength							
Hand grip test (kg)		4	0.18	38%	Fixed-effects model	MD = 2.64, 95%CI (0.02, 5.26)	0.05
30CST (times/30s)		4	0.41	0%	Fixed-effects model	MD = 4.24, 95%CI (2.89, 5.60)	<0.00001
Arm curl test (times/30s)		5	0.09	51%	Random-effects model	MD = 3.82, 95%CI (1.33, 6.30)	0.003
	Control	4	0.14	45%	Random-effects model	MD = 4.23, 95%CI (1.99, 6.48)	0.0002
	Land exercise	2	0.07	69%	Random-effects model	MD = 1.66, 95%CI (−8.77, 12.20)	0.76
	Short-term intervention	3	0.20	38%	Random-effects model	MD = 4.15, 95%CI (0.29, 8.00)	0.04
	Long-term intervention	2	0.03	79%	Random-effects model	MD = 3.40, 95%CI (−1.05, 7.85)	0.13
2. Body composition							
Body fat mass (kg)		4	0.54	0%	Fixed-effects model	MD = −1.35, 95%CI (−2.90, 0.20)	0.09
Body fat percentage (%)		6	0.39	4%	Fixed-effects model	MD = −1.69, 95%CI (−2.26, −1.13)	<0.00001
Secondary outcome							
1. Flexibility							
Sit and reach (cm)		6	<0.0001	81%	Random-effects model	MD = 3.54, 95%CI (0.43, 6.66)	0.03
	Control	5	<0.00001	94%	Random-effects model	MD = 4.26, 95%CI (−0.84, 9.37)	0.10
	Land exercise	2	0.60	0%	Random-effects model	MD = 4.95, 95%CI (3.57, 6.34)	<0.00001
	Short-term intervention	3	0.37	0%	Random-effects model	MD = 7.33, 95%CI (5.93, 8.72)	<0.00001
	Long-term intervention	3	0.91	0%	Random-effects model	MD = 1.98, 95%CI (0.33, 3.64)	0.02
Back scratch (cm)		4	0.50	0%	Fixed-effects model	MD = 2.84, 95%CI (1.94, 3.74)	<0.00001
2. Mobility ability							
Timed up and go test (s)		3	0.04	69%	Random-effects model	MD = −1.09, 95%CI (−1.59, −0.60)	<0.0001
3. Anthropometric indicators							
Body weight (kg)		12	0.76	0%	Fixed-effects model	MD = −0.74, 95%CI (−2.08, 0.60)	0.28
BMI		6	0.99	0%	Fixed-effects model	MD = −0.16, 95%CI (−0.81, 0.48)	0.62
Waist circumference (cm)		4	0.86	0%	Fixed-effects model	MD = −1.56, 95%CI (−4.80, 1.68)	0.35
Hip circumference (cm)		3	0.98	0%	Fixed-effects model	MD = −0.96, 95%CI (−3.59, 1.67)	0.47
Waist-to-hip ratio		5	0.90	100%	Fixed-effects model	MD = 0.00, 95%CI (−0.02, 0.03)	0.76
Arm circumference (cm)		4	0.97	0%	Fixed-effects model	MD = −0.06, 95%CI (−0.79, 0.90)	0.9
Thigh circumference (cm)		4	0.96	0%	Fixed-effects model	MD = −0.04, 95%CI (−1.31, 1.39)	0.96
4. Lipids							
Total cholesterol (mmol/L)		3	0.68	0%	Fixed-effects model	MD = −0.29, 95%CI (−056, −0.02)	0.04
HDL-C (mmol/L)		4	0.65	0%	Fixed-effects model	MD = 0.03, 95%CI (−0.06, 0.12)	0.48
LDL-C (mmol/L)		3	0.65	0%	Fixed-effects model	MD = −0.18, 95%CI (−0.44, 0.09)	0.19
Triglycerides (mmol/L)		4	0.04	64%	Random-effects model	MD = −0.09, 95%CI (−0.33, 0.16)	0.49
	Control	3	0.02	75%	Random-effects model	MD = −0.13, 95%CI (−0.49, 0.23)	0.49
	Land exercise	1	/	/	Random-effects model	MD = 0.00, 95%CI (−0.26, 0.26)	1
	Short-term intervention	2	0.005	87%	Random-effects model	MD = −0.14, 95%CI (−0.82, 0.55)	0.70
	Long-term intervention	2	0.45	0%	Random-effects model	MD = −0.07, 95%CI (−0.25, 0.11)	0.45

N, number of the studies; MD, mean difference; CI, confidence interval; HDL-C, High density lipoprotein cholesterol; LDL-C, Low Density Lipoprotein.

### 3.3 Primary outcomes

#### 3.3.1 Muscle strength

**Figure 2:**
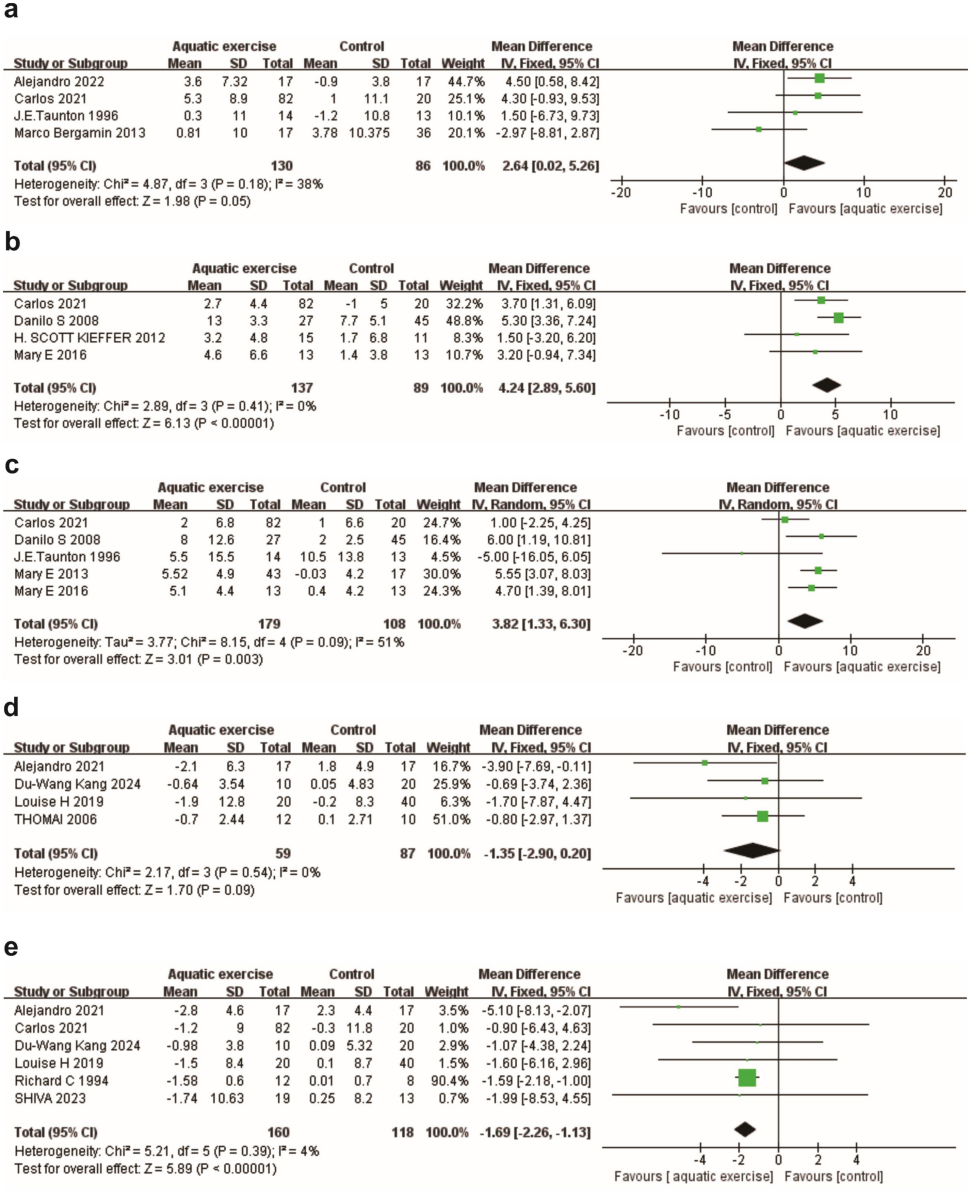
Forest plots for the primary outcomes. **(a)** Hand grip test. **(b)** 30-CST. **(c)** Arm curl test. **(d)** Body fat mass. **(e)** Body fat percentage.

A pooled analysis of four randomized controlled trials (*n* = 216) demonstrated a modest yet significant improvement in hand grip test (kg) within the aquatic exercise group [MD = 2.64 kg, 95% CI (0.02, 5.26), *p* = 0.05] (Figure 2a). Regarding lower limb strength assessed by the 30-CST, four studies (*n* = 226) reported a significant increase in the number of repetitions [MD = 4.24, 95% CI (2.89, 5.60), *p* < 0.00001] (Figure 2b). Additionally, five studies (*n* = 287) indicated enhanced upper limb strength, measured by the Arm Curl Test (reps/30s), favoring the aquatic exercise group [MD = 3.82 repetitions, 95% CI (1.33, 6.30), *p* = 0.003; *I^2^* = 51%] (Figure 2c).

Given the heterogeneity observed, we conducted subgroup analyses based on the mode and duration of the intervention. The findings indicated a significant advantage for the aquatic exercise group compared to the control group [MD = 4.23, 95% CI (1.99, 6.48), *p* = 0.0002] (Supplementary Figure S2a), although no significant difference was observed when compared to the land-based exercise group [MD = 1.66, 95% CI (−8.87, 12.20), *p* = 0.76] (Supplementary Figure S2a). Previous research has established a time point that differentiates between short- and long-term exercise interventions, categorizing them as less than 12 weeks or greater than 12 weeks in duration (56). We repeated the subgroup analyses based on intervention duration and found that short-term interventions (<12 weeks) [MD = 4.15, 95% CI (0.29, 8.00), *p* = 0.04] (Supplementary Figure S3a) were more effective than long-term interventions [MD = 3.40, 95% CI (−1.05, 7.85), *p* = 0.13] (Supplementary Figure S3a).

#### 3.3.2 Body composition

The pooled analysis revealed no significant difference between the groups in terms of Absolute Body Fat Mass (kg) [MD = −1.35, 95% CI (−2.90, 0.20), *p* = 0.09] (Figure 2d). In contrast, regarding Body Fat Percentage (%), the analysis of six studies (*n* = 278) indicated a clinically significant decrease in the aquatic exercise group [MD = −1.69, 95% CI (−2.26, −1.13), *p* < 0.00001] (Figure 2e).

### 3.4 Secondary outcomes

#### 3.4.1 Lipid profiles

**Figure 3:**
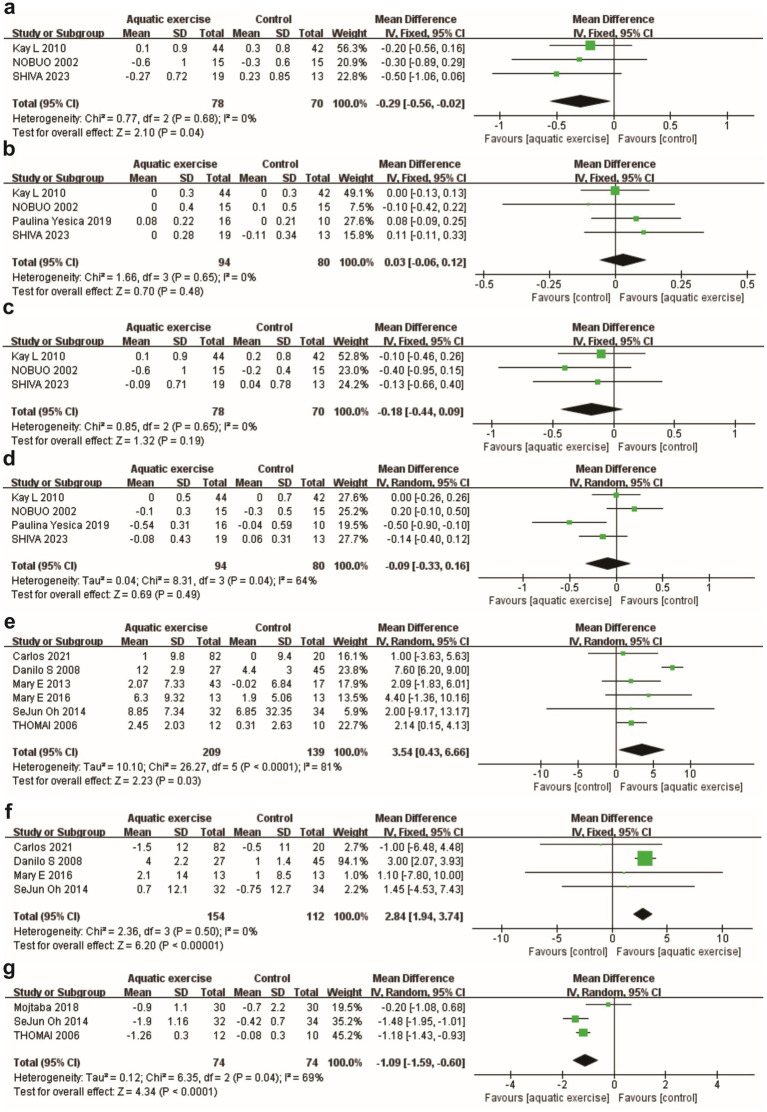
Forest plots for the secondary outcomes. **(a)** Total cholesterol; **(b)** HDL-C; **(c)** LDL-C; **(d)** Triglycerides; **(e)** Sit and reach; **(f)** Back scratch; **(g)** Timed Up and Go (TUG).

The pooled analysis of three studies (*n* = 148) revealed a significant reduction in total cholesterol levels (mmol/L) in the aquatic exercise group [MD = −0.29, 95% CI (−0.56, −0.02), *p* = 0.04] (Figure 3a). However, no significant changes were observed in HDL-C, LDL-C, or Triglyceride levels (mmol/L) [HDL-C: MD = 0.03, 95% CI (−0.06, 0.12), *p* = 0.48; LDL-C: MD = −0.18, 95% CI (−0.44, 0.09), *p* = 0.19; triglyceride: MD = −0.09, 95% CI (−0.33, 0.16), *p* = 0.49; *I^2^* = 64%] (Figures 3b–d). Subgroup analyses based on types of the intervention [control group: MD = −0.13, 95% CI (−0.49, 0.23), *p* = 0.49; land exercise: MD = 0.00, 95% CI (−0.26, 0.26), *p* = 1.00] (Supplementary Figure S2b) and duration [short-term: MD = −0.14, 95% CI (−0.82, 0.55), *p* = 0.70; long-term: MD = −0.07, 95% CI (−0.25, 0.11), *p* = 0.45] (Supplementary Figure S3b) also confirmed no significant effects.

#### 3.4.2 Flexibility

Results from six studies (n = 348) indicated that aquatic exercise significantly improved flexibility, as measured by Sit and reach test (cm) [MD = 3.54, 95% CI (0.43, 6.66), *p* = 0.03; *I^2^* = 81%] (Figure 3e). Subgroup analyses revealed a more pronounced improvement in flexibility for land-based exercise compared to no exercise [MD = 4.95, 95% CI (3.57, 6.34), *p* < 0.00001] (Supplementary Figure S2c). Although aquatic exercise also showed a trend toward improvement, the evidence was less robust than that for the land-based exercise control group [MD = 4.26, 95% CI (−0.84, 9.37), *p* = 0.10] (Supplementary Figure S2c). When examining intervention duration, short-term interventions yielded greater improvements [MD = 7.33, 95% CI (5.93, 8.72), *p* < 0.00001] compared to long-term interventions [MD = 1.98, 95% CI (0.33, 3.64), *p* = 0.02] (Supplementary Figure S3c). Meta-regression results indicated that the duration and types of intervention in the control group were key factors influencing the source of heterogeneity (Supplementary Table S2). Regarding back scratch (cm), the inclusion of four studies (n = 266) demonstrated a significant improvement in the aquatic exercise group [MD = 2.84 cm, 95% CI (1.94, 3.74), *p* < 0.00001] (Figure 3f).

#### 3.4.3 Mobility ability

The findings of three studies (*n* = 148) indicated a reduction in TUG time among participants engaging in aquatic exercise [MD = −1.09, 95% CI (−1.59, −0.60), *p* < 0.0001] (Figure 3g).

#### 3.4.4 Anthropometric indicators

**Figure 4:**
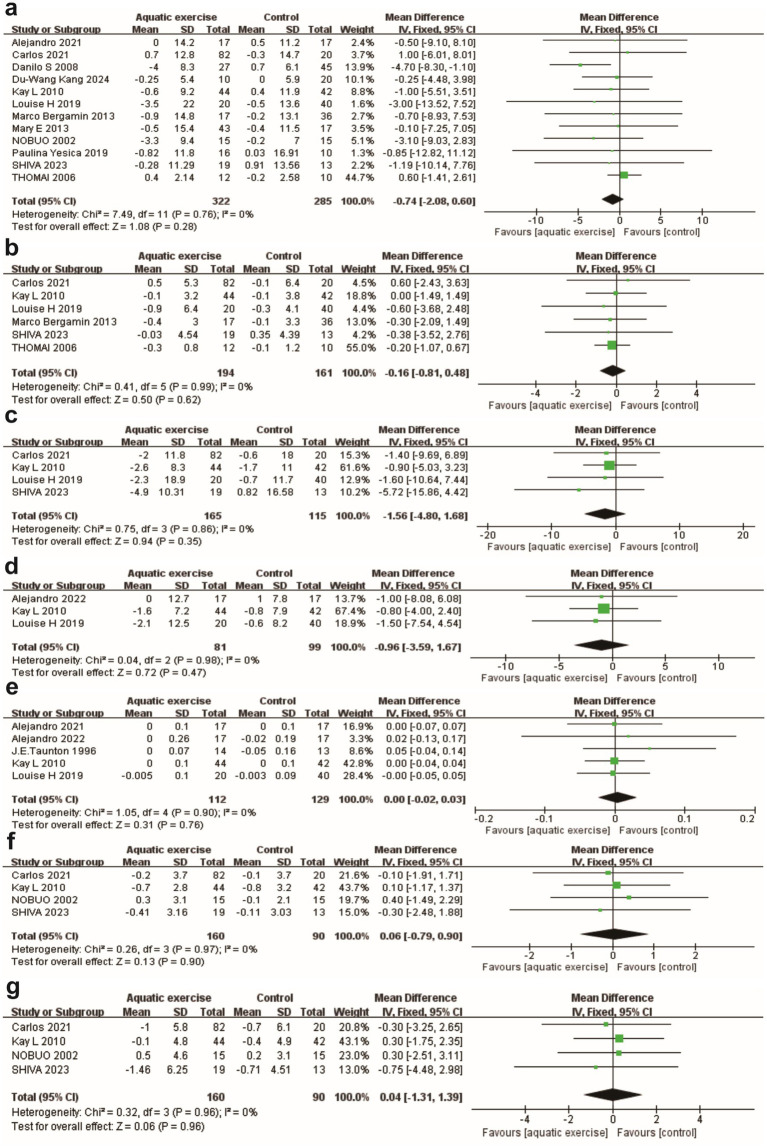
Forest plots for the secondary outcomes continued. **(a)** Body weight. **(b)** BMI. **(c)** Waist circumference. **(d)** Hip circumference. **(e)** Waist-to-hip ratio. **(f)** Arm circumference. **(g)** Thigh circumference.

Pooled analyses of anthropometric indicators showed no significant differences between aquatic exercise and control groups across assessed variables. Specifically, no significant changes were observed in body weight [MD = −0.74 kg, 95% CI (−2.08, 0.60), *p* = 0 0.28; *I^2^* = 0%] (Figure 4a), BMI [MD = −0.16, 95% CI (−0.81, 0.48), *p* = 0.62; *I^2^* = 12%] (Figure 4b), waist circumference (cm) [MD = −1.56, 95% CI (−4.80, 1.68), *p* = 0.35; *I^2^* = 0%] (Figure 4c), hip circumference (cm) [MD = −0.96, 95% CI (−3.59, 1.67), *p* = 0.47; *I^2^* = 0%] (Figure 4d), and waist-to-hip ratio [MD = 0.00, 95% CI (−0.02, 0.03), *p* = 0.76; *I^2^* = 0%] (Figure 4e). Similarly, circumference measurements for the arm and thigh (cm) did not reveal significant changes [arm: MD = 0.06, 95% CI (−0.97, 0.90), *p* = 0.90; thigh: MD = 0.04, 95% CI (−1.31, 1.39), *p* = 0.96] (Figures 4f,g).

### 3.5 Sensitivity analysis and funnel plot

Sensitivity analysis (Supplementary Figures S4a–h) demonstrated that the outcomes for 30-CST and Body Fat Percentage were robust and consistent. Conversely, results for the Arm Curl Test, Back Scratch Test, total cholesterol, hand grip test, sit and reach, and TUG exhibited variability following the sequential exclusion of individual studies. Funnel plot results regarding publication bias are presented in Supplementary Figures S5a–j, S6a–j.

## 4 Discussion

The primary objective of this study was to comprehensively evaluate the effects of aquatic exercise on muscle strength, body composition, and physical and metabolic health in healthy older adults. Synthesizing data from 19 randomized controlled trials involving 866 participants, our findings demonstrate that aquatic exercise significantly improves lower limb muscle strength, flexibility, and mobility. Notably, reductions in body fat percentage and total cholesterol levels were also observed, although changes in other anthropometric and lipid parameters were not statistically significant. Sensitivity analyses confirmed the robustness of improvements in 30-CST performance and body fat percentage, whereas outcomes such as upper limb strength and hand grip showed greater variability.

In this study, the 30 CST was used as the primary indicator for lower limb muscle strength. The results revealed that participants in the aquatic exercise group performed significantly better than those in the control group. Sensitivity analyses further supported the stability of these findings, affirming the effectiveness of aquatic exercise in enhancing lower limb strength in older adults. These findings align with previous systematic reviews demonstrating that the resistance provided by water is sufficient to elicit strength gains in the lower extremities of older adults (29). Upper limb and wrist strength were assessed using the arm curl and hand grip tests, respectively. Research by Prado et al. (30) suggests that aquatic exercise can notably enhance grip strength, particularly when equipment is utilized. Nonetheless, sensitivity analyses excluding devices show no discernible difference between aquatic exercise and the control group (30). Consistent with these findings, our study demonstrates that although the aquatic exercise group displayed significant enhancements in these metrics, sensitivity analyses indicate that the outcomes were not statistically robust upon exclusion of specific studies. This inconsistency may be attributed to heterogeneity in the exercise types, intensity, frequency, and target muscle groups across the included studies. Therefore, further research with standardized intervention protocols is warranted to establish the effects of aquatic exercise on upper limb and wrist strength.

Muscle strength is closely associated with physical function, and previous studies have demonstrated strong correlations between muscular strength, gait speed, and reaction time (60, 61). In this context, the HDL-C test serves as a widely used functional assessment tool in older adults, indirectly reflecting overall muscular performance (62). Our analysis revealed that participants in the aquatic exercise group showed significantly reduced TUG times compared to the control group, suggesting improved mobility capacity. This is consistent with prior study indicating positive effects of aquatic exercise on functional mobility (63). However, sensitivity analysis indicated limited stability of this result, highlighting the need for further studies to validate the effect.

Flexibility, like muscle strength, is a key determinant of functional independence in older adults. It influences performance in daily activities and has been shown to improve in response to certain resistance training regimens (64). In our study, aquatic exercise led to significant improvements in both the sit-and-reach and back scratch tests compared to control conditions. These findings remained generally consistent across sensitivity analyses. Our results reinforce earlier evidence suggesting that the warmth and buoyancy of water create an optimal environment for increasing joint range of motion and soft tissue extensibility (65). Meta-regression further identified exercise modality and intervention duration as potential sources of heterogeneity, particularly in the sit-and-reach results. Subgroup analysis indicated that longer-term interventions tended to produce more substantial improvements. Collectively, these findings suggest that aquatic exercise is especially effective in enhancing flexibility among older adults.

Anthropometric indicators are generally categorized into three types: longitudinal (e.g., height, body length), transverse (e.g., head circumference, chest circumference), and weight-related parameters (e.g., body weight, skinfold thickness) (66). These indicators provide a comprehensive assessment of an individual’s nutritional status and are often used in evaluating the risk of chronic and cardiovascular diseases (67, 68). In our meta-analysis, no significant differences were observed between the aquatic exercise and control groups in terms of body weight and BMI. This may be due to the heterogeneous nature of the control groups, which included both land-based exercise and non-exercise conditions. Variations in intervention intensity across studies may also have contributed to these mixed findings. Additionally, differences in physiological responses between aquatic and terrestrial exercise, including variations in energy expenditure, metabolic rate, and recruited muscle groups, could influence outcomes related to energy balance and body composition (69, 70). For other anthropometric measures such as waist circumference, hip circumference, and waist-to-hip ratio, our results also showed no statistically significant differences. Interestingly, the mean difference tended to favor aquatic exercise for most measures, except for arm and leg circumferences. Some evidence suggests that the effect of swimming on waist circumference may be more pronounced in the short term, while effects on hip and calf circumference may manifest over both short and long durations. These variations could be attributed to the distinct muscle group activation patterns in swimmers versus walkers (71).

Compared to anthropometric measurements, which serve as relatively indirect indicators of health risk, changes in body composition offer a more direct and informative reflection of metabolic and physiological health status. In the current study, participants in the aquatic exercise group exhibited significantly greater reductions in body fat percentage and total cholesterol compared to the control group. Sensitivity analysis further confirmed the stability of the fat percentage reduction, reinforcing the reliability of this finding. However, changes in HDL-C, LDL-C, and triglyceride levels were not statistically significant. This may be attributed to differences in exercise intensity, as previous studies have shown that only high-intensity aquatic activity, such as vigorous swimming, can meaningfully improve HDL-C levels, particularly in young, trained individuals (72). Additionally, intervention duration appears to play a crucial role: a 12-month aquatic exercise program was more effective at suppressing lipid accumulation than a 6-month program (44). Overall, favorable alterations in lipid profiles appear to depend on both the intensity and the duration of aquatic exercise interventions (73).

This review is subject to several limitations. First, regarding evidence quality and sample characteristics, the risk of bias varied among the included studies (primarily due to insufficient reporting of randomization procedures in some trials), and sample sizes were generally small. These factors warrant a contextualized interpretation of the findings rather than diminishing the overall trend. Second, inherent limitations regarding clinical heterogeneity and data availability existed in the included literature. The interventions encompassed eight diverse aquatic modalities with varying intensities and frequencies, and there was a predominance of female participants. These variations limited the feasibility of performing extensive stratified analyses based on gender or specific intervention modes. Similarly, concerning outcome data, although we aimed to investigate the broad domain of “body composition,” the pooled results were predominantly restricted to fat mass and body fat percentage due to insufficient reporting of other components (e.g., lean mass) in primary studies. Thus, our findings under this heading primarily reflect changes in body fat. Third, methodological heterogeneity was observed, particularly regarding metabolic outcomes. Although random-effects models were employed to account for statistical heterogeneity, variations in measurement protocols (e.g., lipid assay kits and fasting states) across original trials may influence the comparability of absolute values.

Future directions to address these limitations and enhance the generalizability of findings, future RCTs should prioritize specific study design features. First, regarding intervention standardization, researchers should explicitly report the FITT principles (Frequency, Intensity, Time, and Type) and consider using uniform intensity monitoring methods to reduce methodological heterogeneity. Second, there is an urgent need to improve sex balance by actively recruiting male participants, allowing for potential sex-specific analyses of aquatic exercise efficacy. Finally, rigorous reporting of adherence and compliance rates is essential to accurately assess the dose–response relationship and the feasibility of long-term interventions.

## 5 Conclusion

This meta-analysis demonstrates that aquatic exercise is effective in improving lower limb strength, flexibility, and mobility in older adults. A moderate reduction in body fat percentage and total cholesterol was also observed, although no significant changes were found in other lipid or anthropometric outcomes. These findings support aquatic exercise as a low-impact, functional training option for aging populations, while highlighting the need for further research on its effects on metabolic health and body composition.

## Data Availability

The original contributions presented in the study are included in the article/Supplementary material, further inquiries can be directed to the corresponding author.
