# Correction: Effects of aquatic exercise on improving body composition and muscle strength in the older adults: a systematic review and meta-analysis of randomized controlled trials

**DOI:** 10.3389/fpubh.2026.1814916

**Published:** 2026-03-06

**Authors:** Yuan Gao, Wenze Deng, Qiancheng Zeng, Yichen Liu, Xiaofu Tang, Sitian Fang, Liang Hao, Hongbo Li

**Affiliations:** 1 Nanchang County People's Hospital, Nanchang, Jiangxi, China; 2 Department of Orthopedics, The Second Affiliated Hospital, Jiangxi Medical College, Nanchang University, Nanchang, Jiangxi, China; 3 School of Nursing, Jiangxi Medical College, Nanchang University, Nanchang, Jiangxi, China; 4 Huankui Academy, Jiangxi Medical College, Nanchang University, Nanchang, Jiangxi, China; 5 Department of Clinical Medicine, The First Clinical College of Nanchang University, Nanchang, Jiangxi, China; 6 The Second Affiliated Hospital, Jiangxi Medical College, Nanchang University, Nanchang, China; 7 Institute of Orthopedics of Jiangxi Province, Nanchang, Jiangxi, China; 8 Jiangxi Provincial Key Laboratory of Spine and Spinal Cord Disease, Nanchang, Jiangxi, China; 9 Institute of Minimally Invasive Orthopedics, Nanchang University, Nanchang, Jiangxi, China

**Keywords:** aging, aquatic exercise, body composition, meta-analysis, muscle strength, systematic review

The order of affiliations in the original article was incorrect. The corresponding author's primary affiliation “Nanchang County People's Hospital, Nanchang, Jiangxi, China”, previously listed as affiliation 9, should be listed as Affiliation 1 and linked to the corresponding author “Hongbo Li”. All other affiliations were renumbered accordingly.

The original version of this article has been updated.

