# Supplementary material for: Effects of aquatic exercise on improving body composition and muscle strength in the older adults: a systematic review and meta-analysis of randomized controlled trials
Source: Front Public Health. 2026 Feb 10;13:1726568. doi: 10.3389/fpubh.2025.1726568 (PMC12930738; doi:10.3389/fpubh.2025.1726568)
Supplement: Supplementary file 1 [file Supplementary_file_1.docx]

**Supplementary materials**


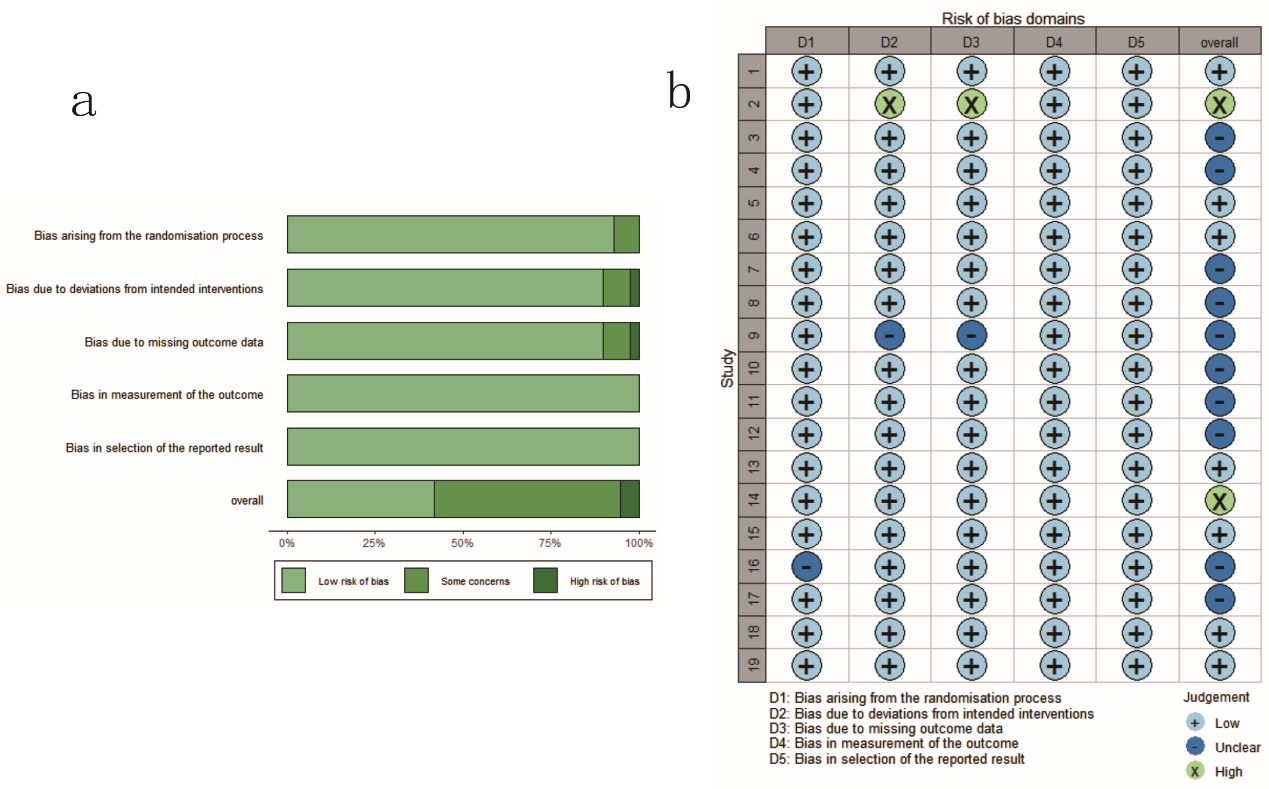


**Supplementary Figure 1. sensitivity analysis.** (a) Risk of bias graph of each included study. (b) Risk of bias summary of each included study.


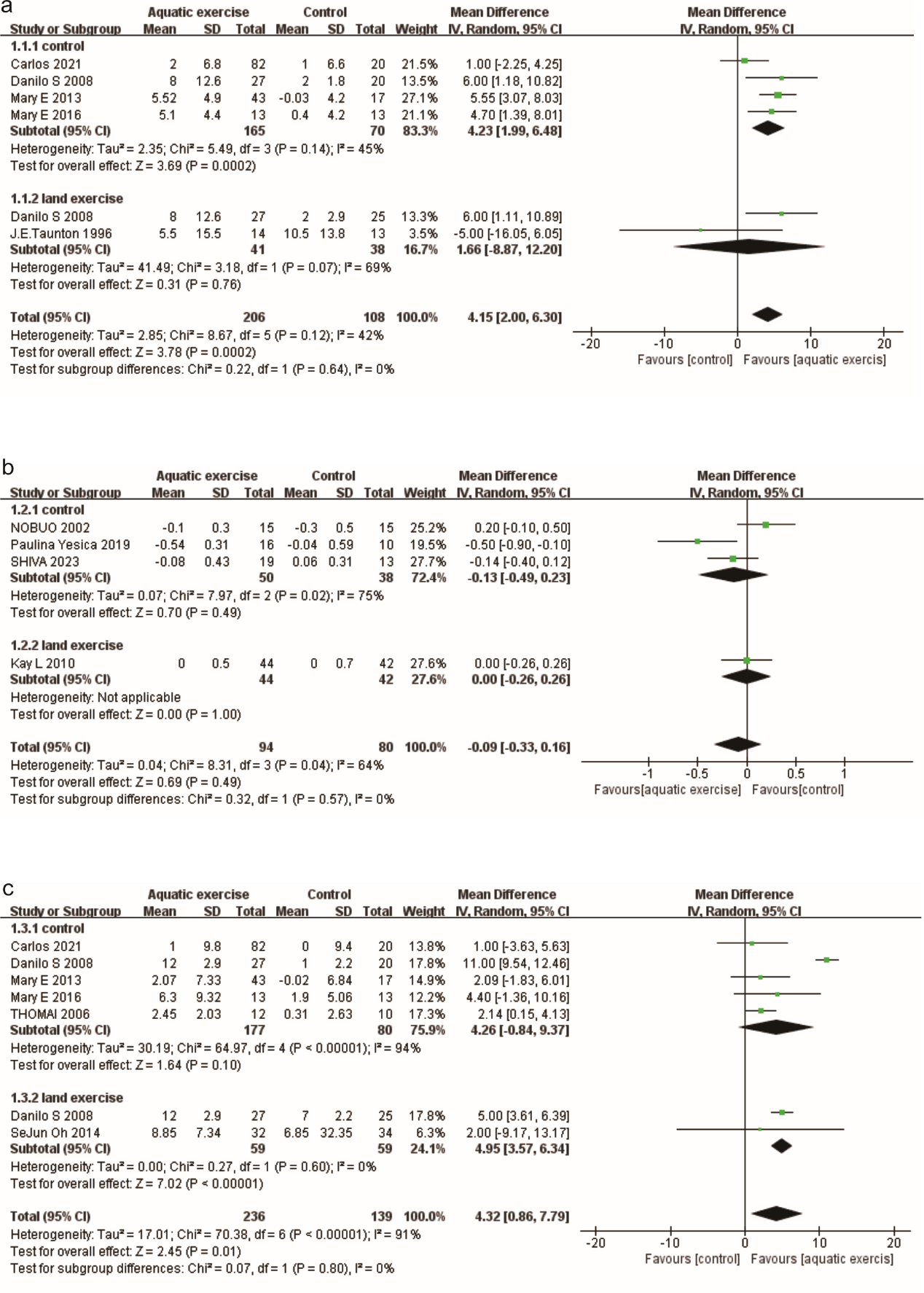


**Supplementary Figure 2. Forest plots for subgroup analysis of intervention types in the control group. (**a). Arm curl test. (b)Triglyceride. (c). Sit and reach.


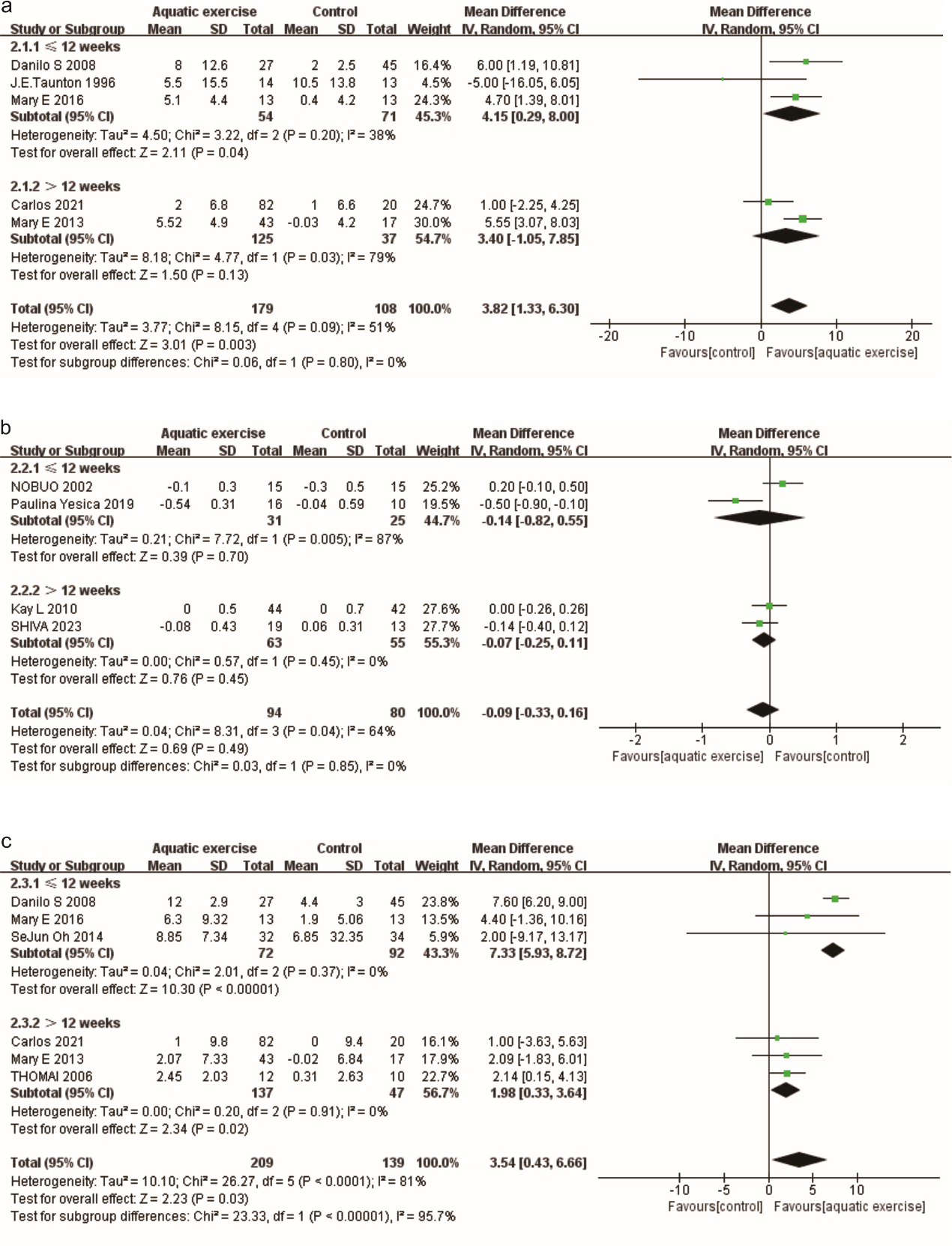


**Supplementary Figure 3. Forest plots for subgroup analysis of duration.** (a). Arm curl test. (b). Triglyceride. (c). Sit and reach.

**Supplementary Figure 4. Sensitivity analysis.**


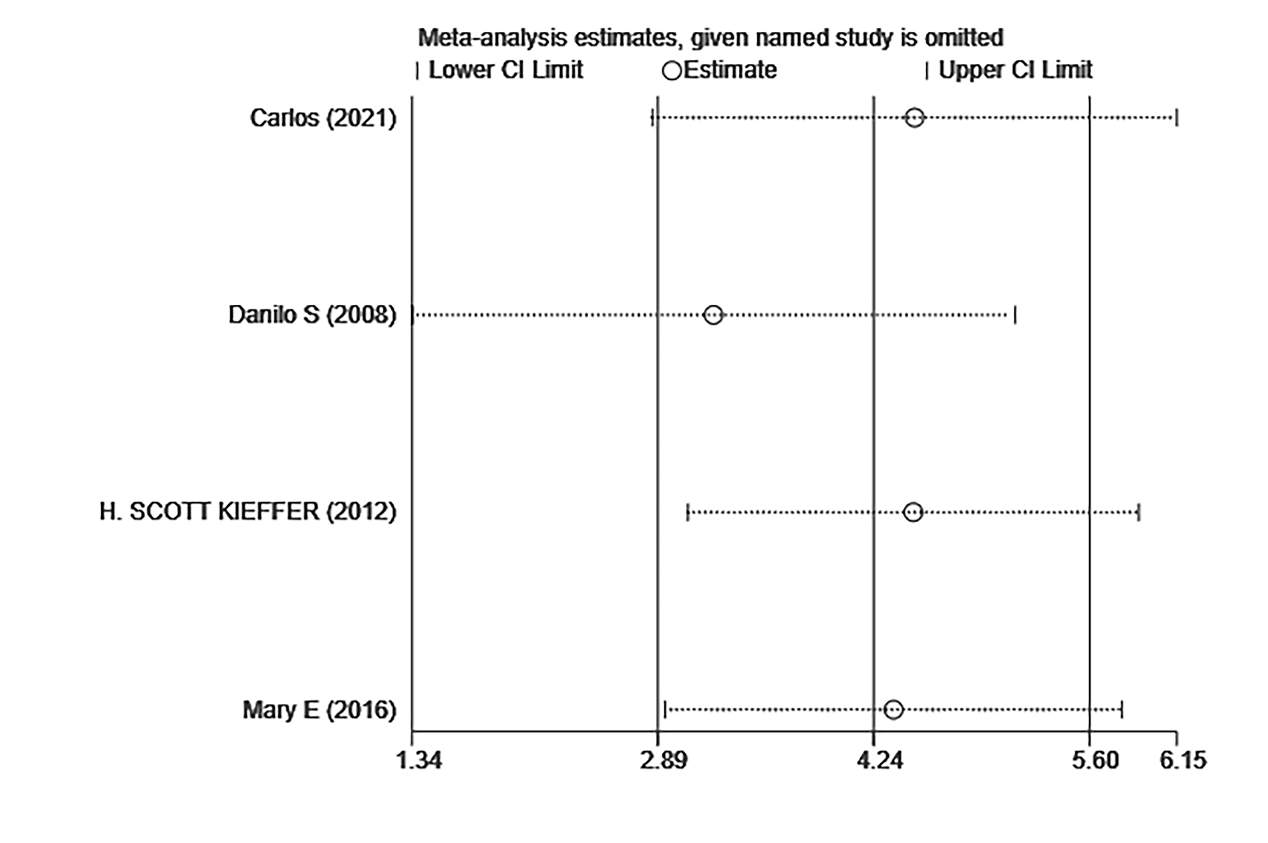


**Fig.s4a. 30-second chair stand test (30-CST).**


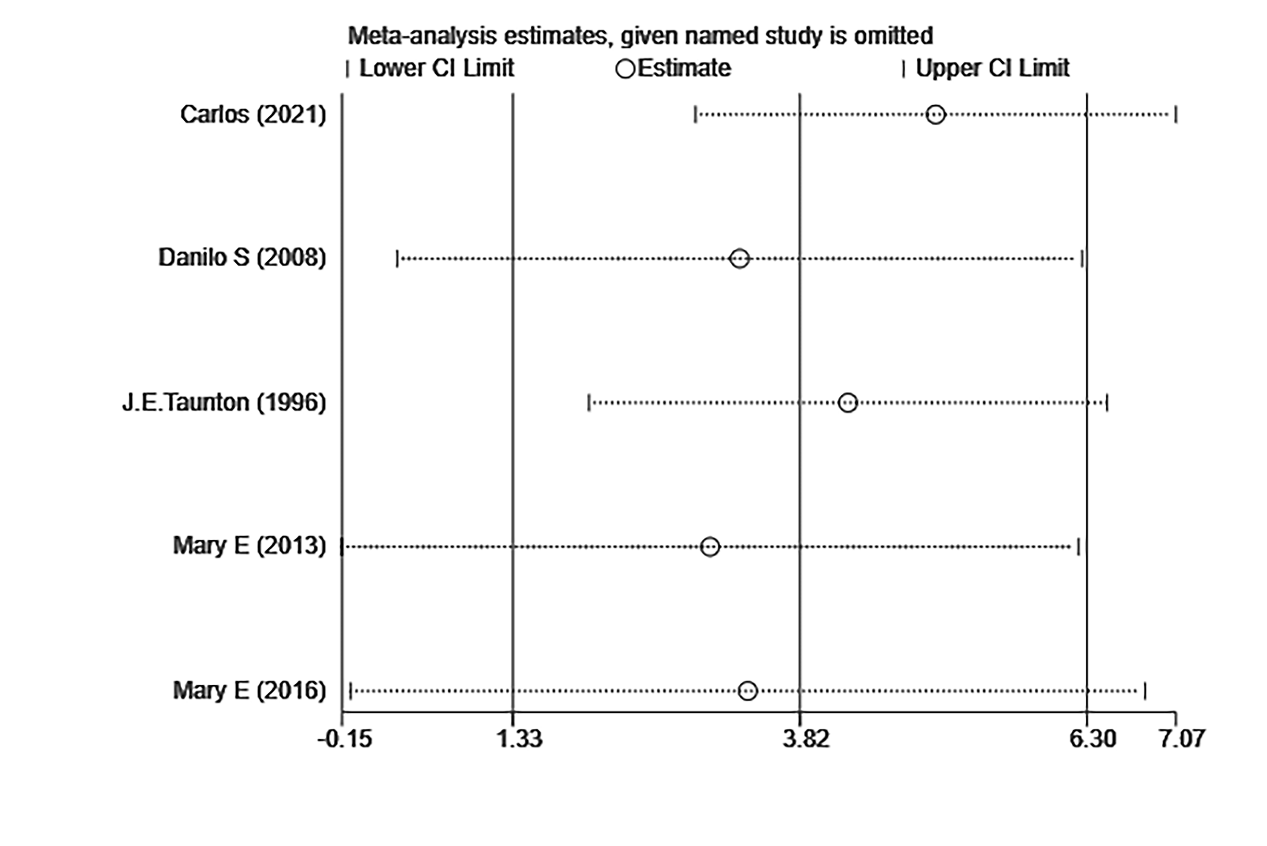


**Fig.s4b. Arm curl test.**


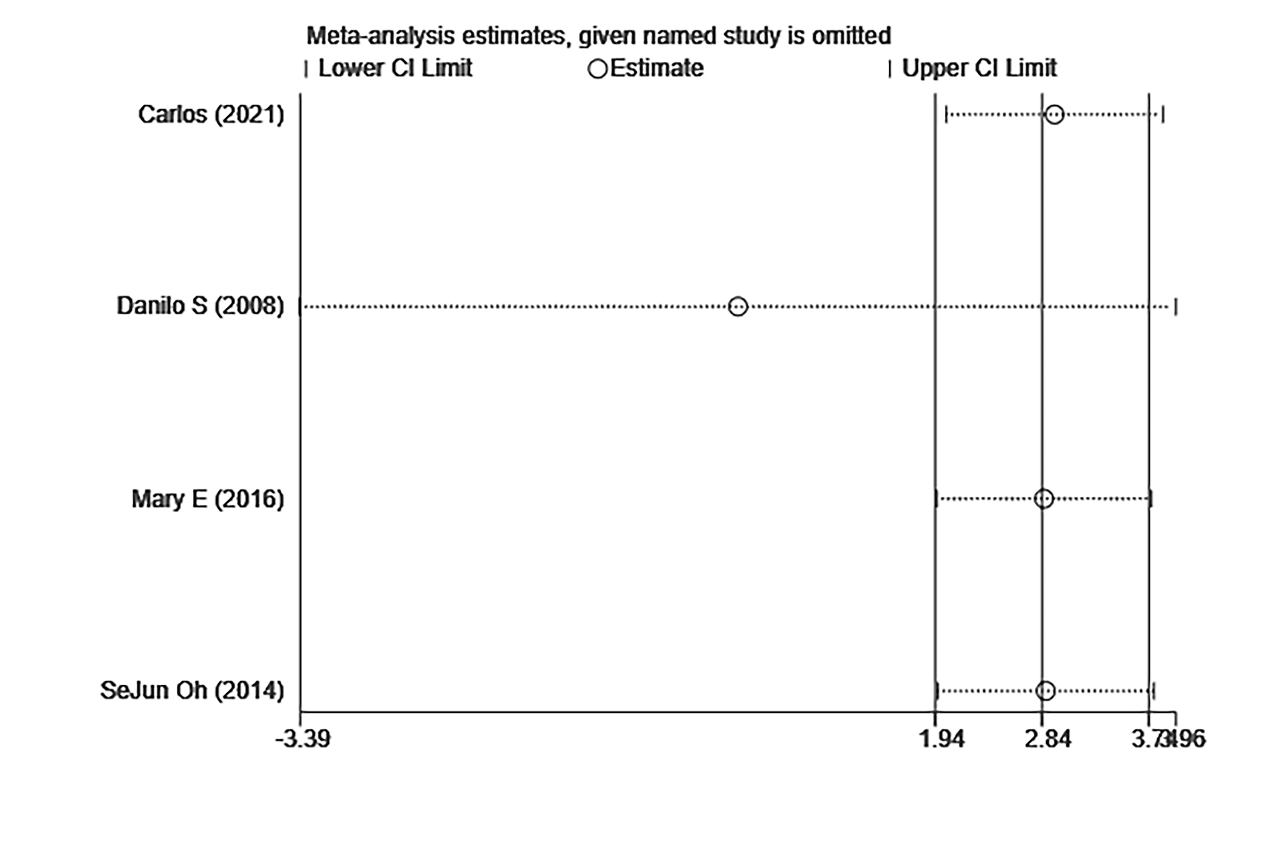


**Fig.s4c. Back scratch.**


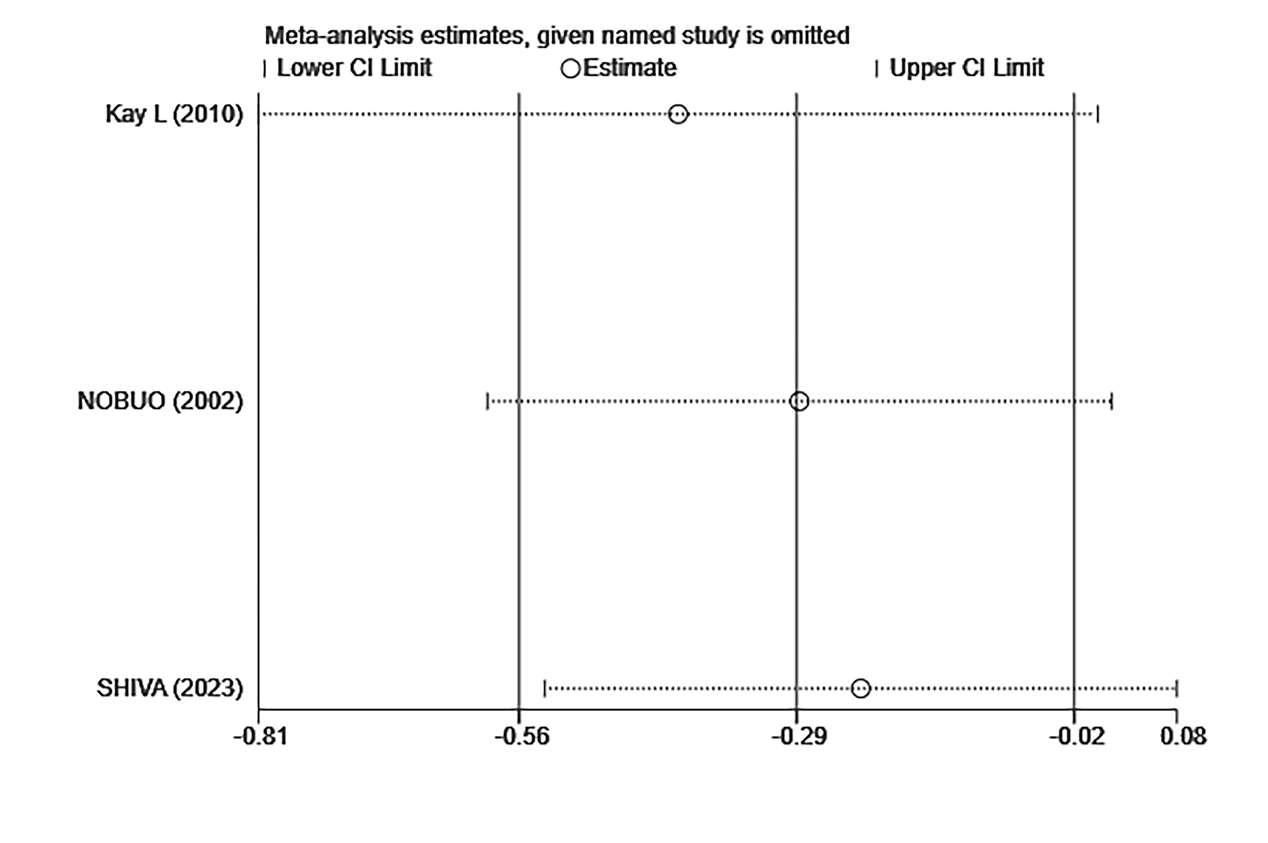


**Fig.s4d. Cholesterol.**


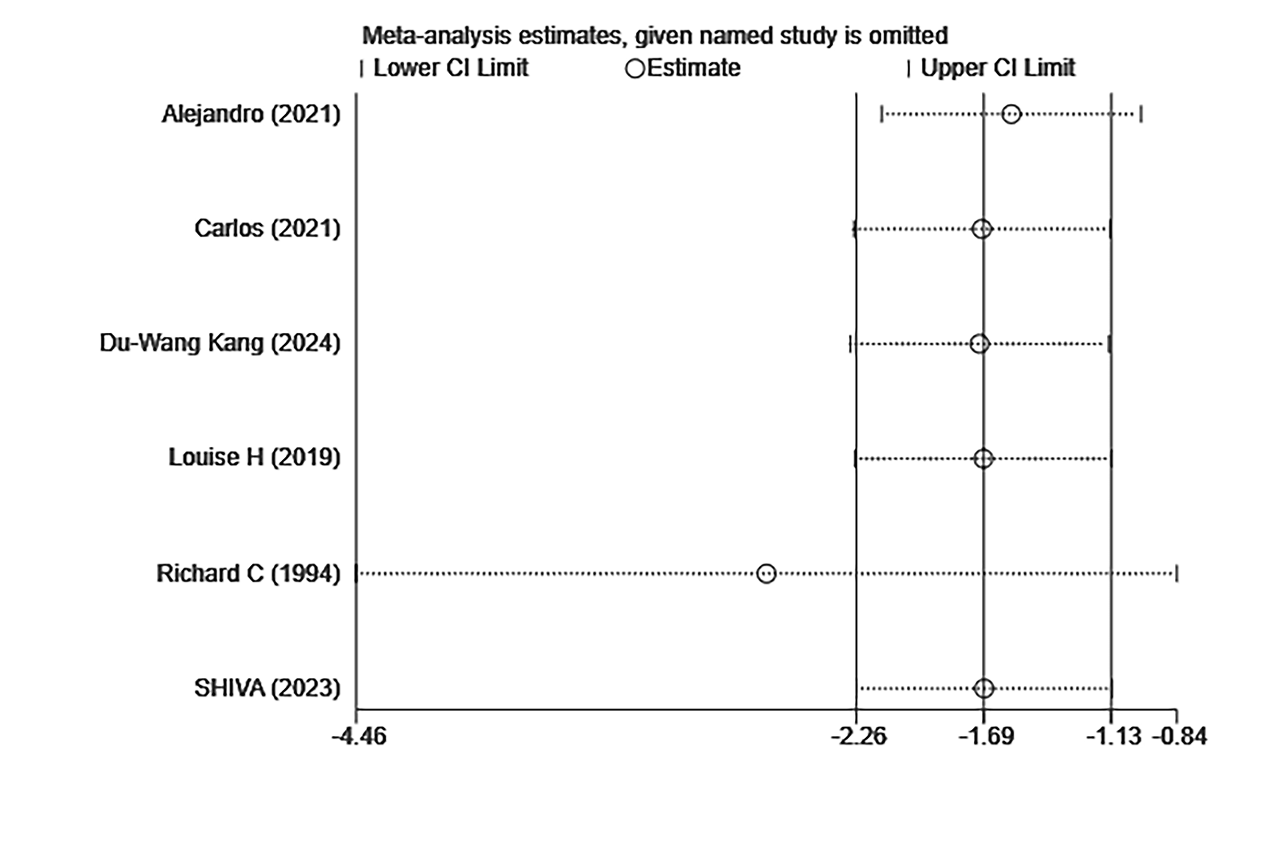


**Fig.s4e. Body Fat Percentage.**


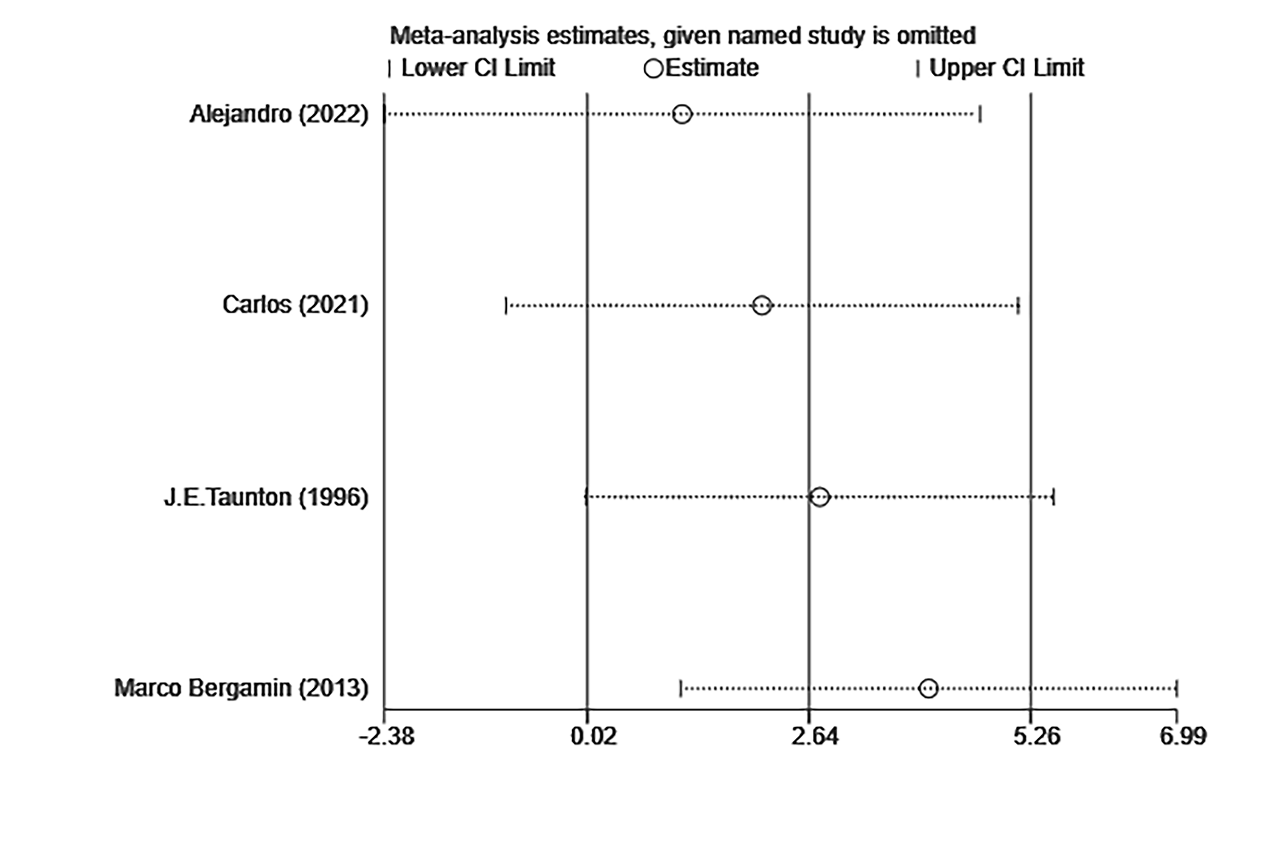


**Fig.s4f. Hand grip test.**


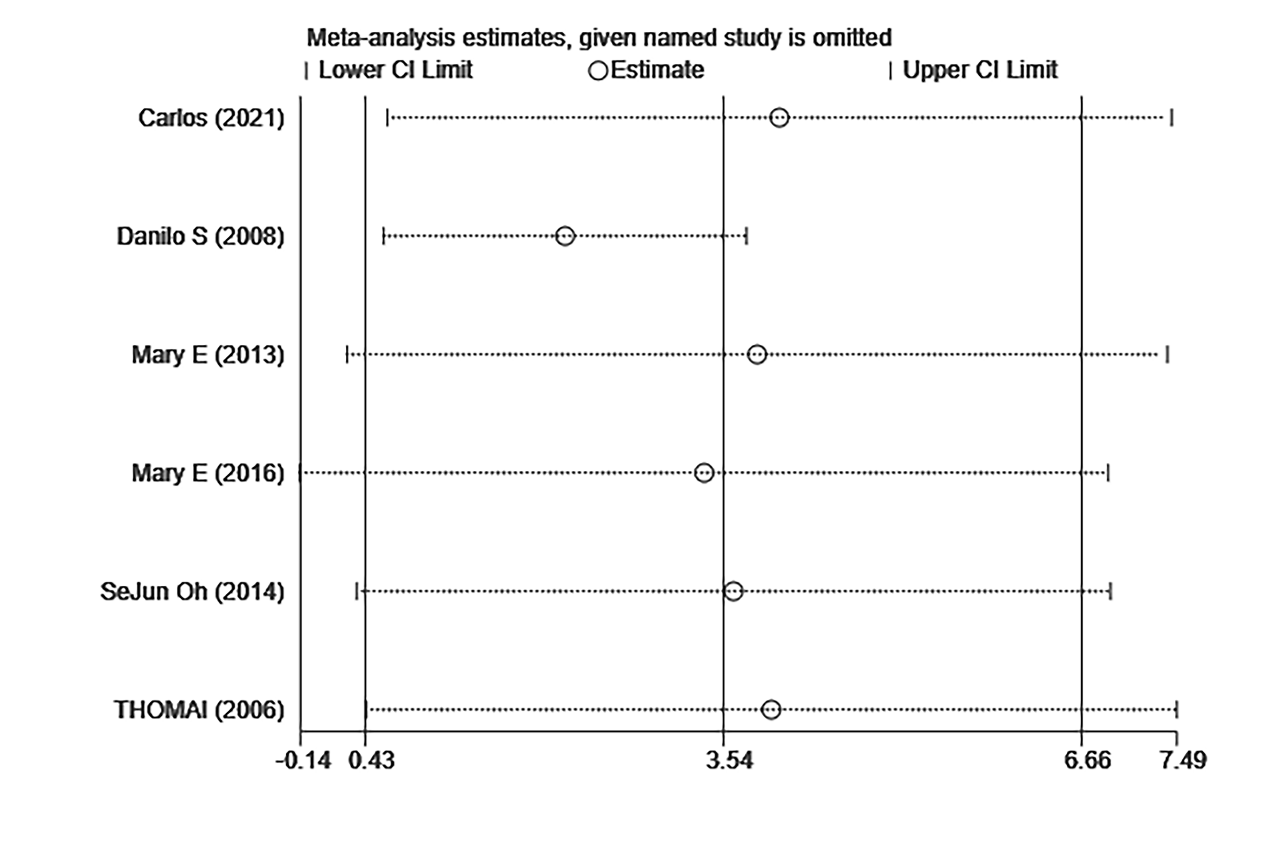


**Fig.s4g. Sit and reach.**


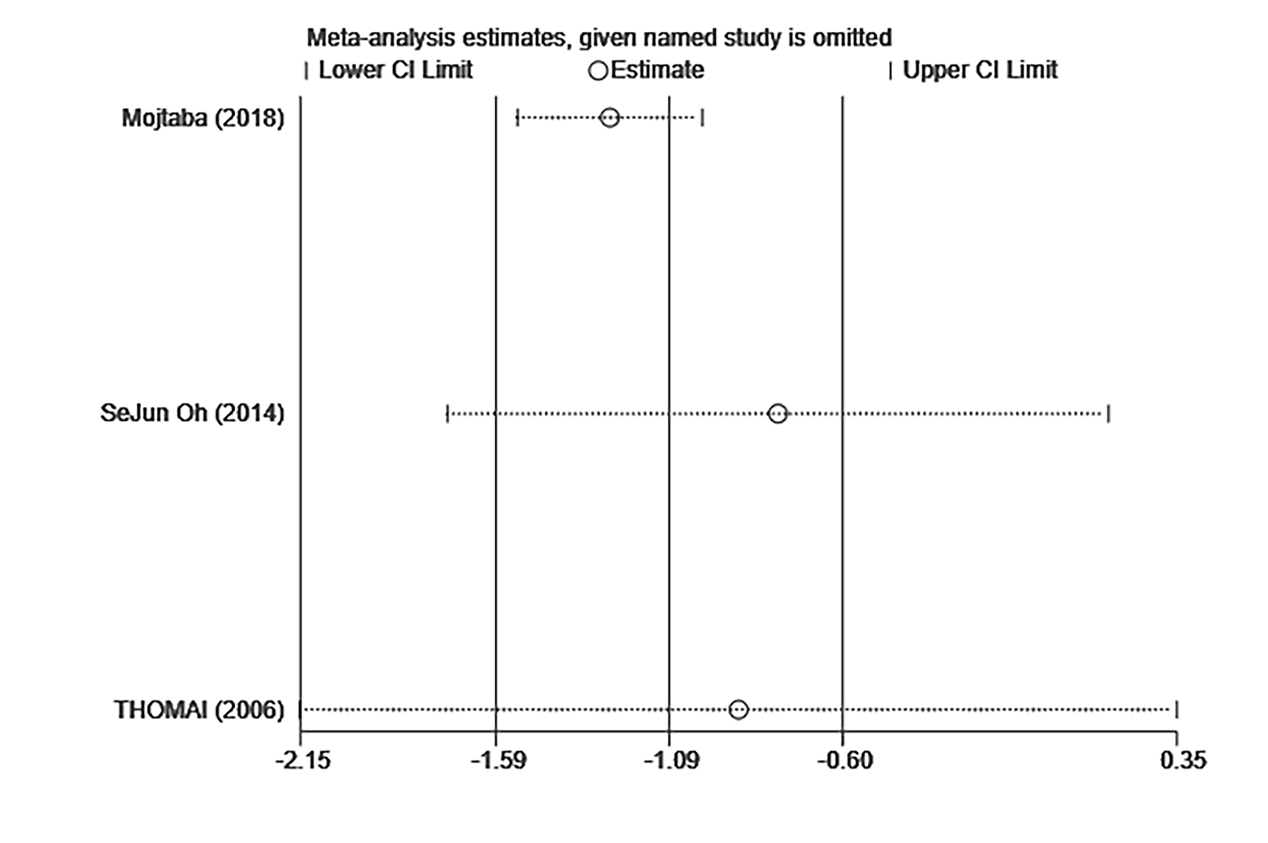


**Fig.s4h. Timed Up and Go (TUG).**

**Supplementary Figure 5. Funnel plot of primary outcomes.**


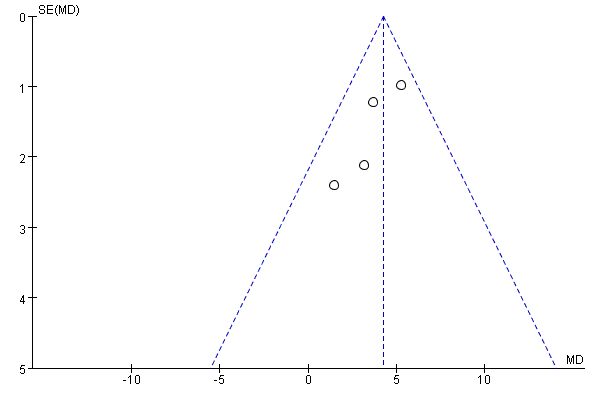


**Fig.s5a. 30-second chair stand test (30-CST).**


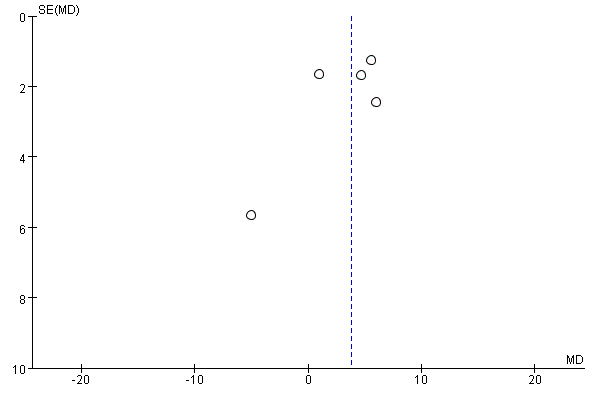


**Fig.s5b. Arm curl test.**


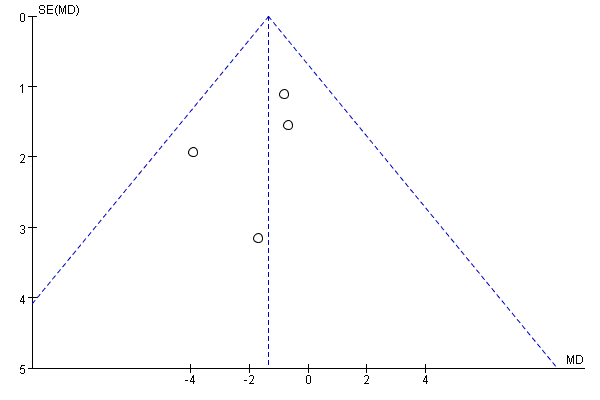


**Fig.s5c.Body fat mass.**


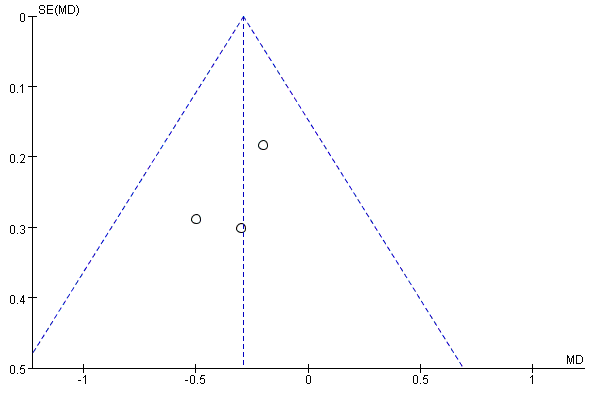


**Fig.s5d. Cholesterol.**


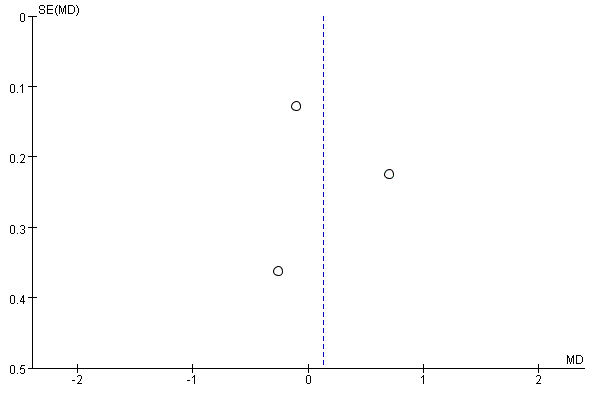


**Fig.s5e. Fasting glucose.**


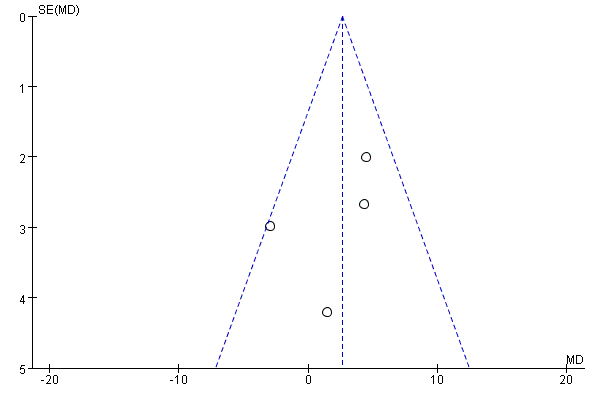


**Fig.s5f. Hand grip test.**


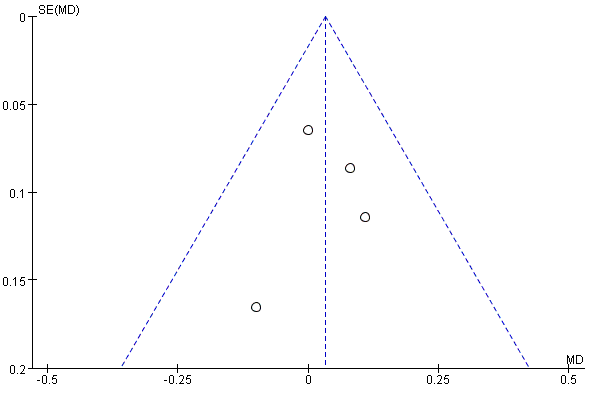


**Fig.s5g. High density lipoprotein cholesterol (HDL-C).**


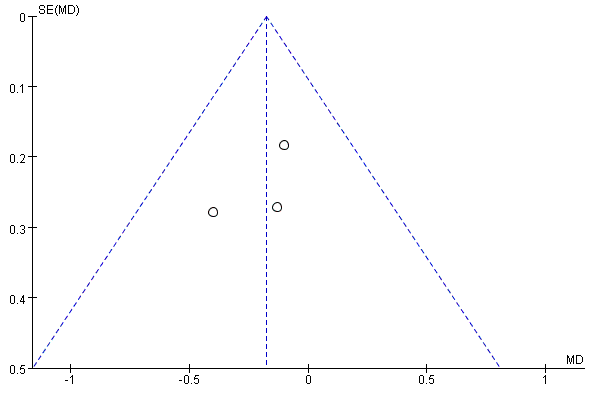


**Fig.s5h. Low Density Lipoprotein (LDL-C).**


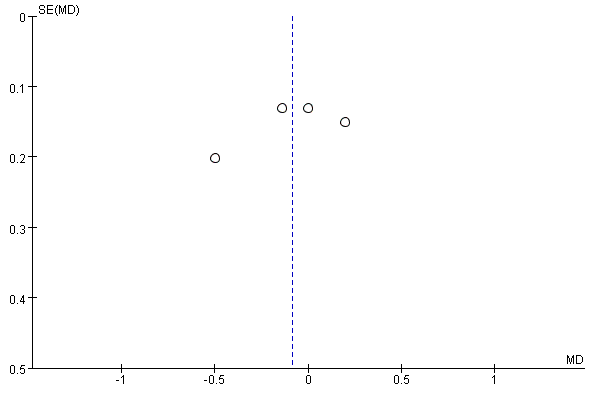


**Fig.s5i. Triglyceride.**


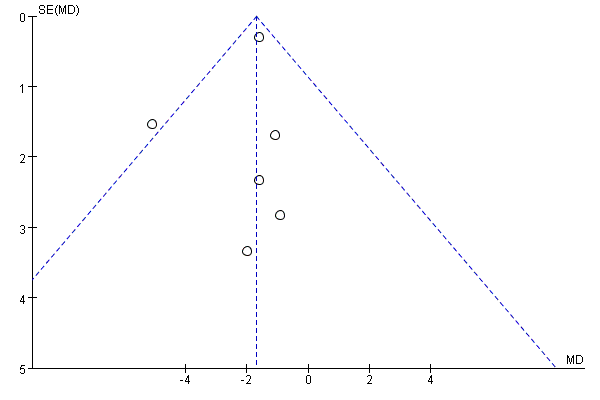


**Fig.s5j.** **Body Fat Percentage.**

**Supplementary Figure 6. Funnel plot of secondary outcomes.**


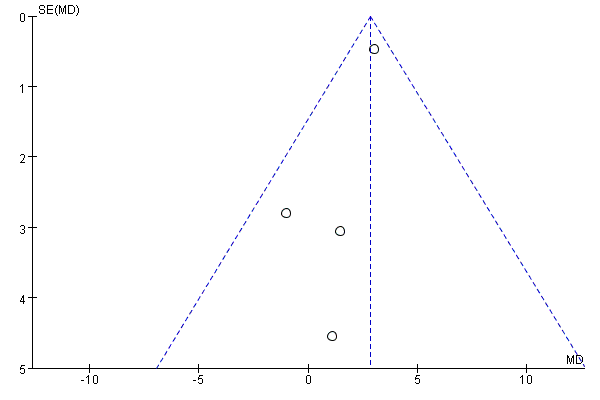


**Fig.s6a. Back scratch.**


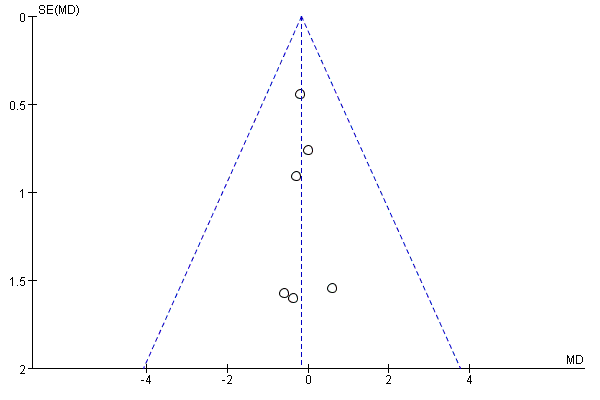


**Fig.s6b. BMI.**


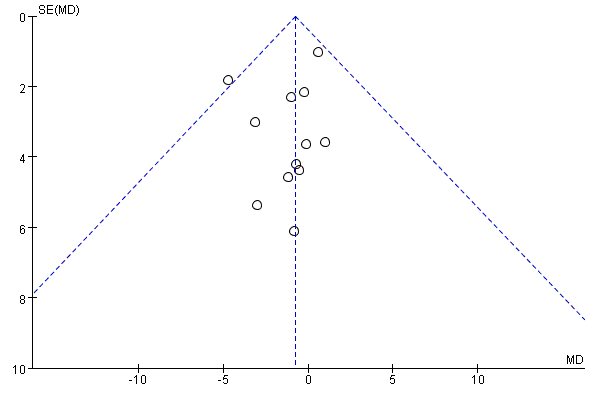


**Fig.s6c. Body weight.**


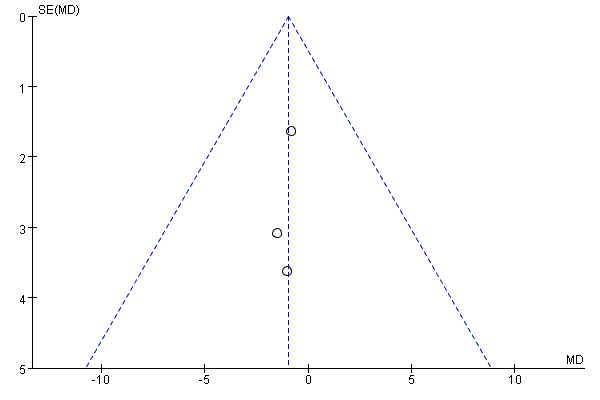


**Fig.s6d. Hip circumference.**


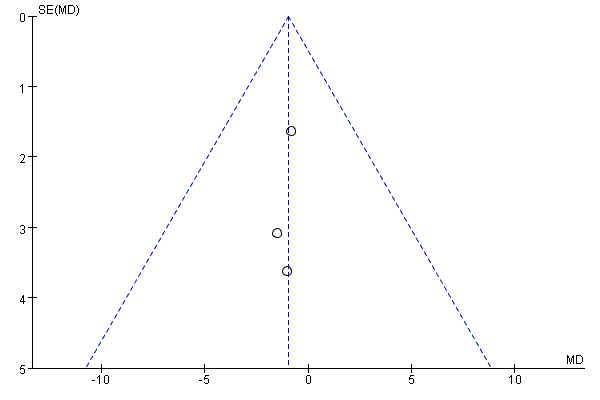


**Fig.s6e. Thigh circumference.**

**
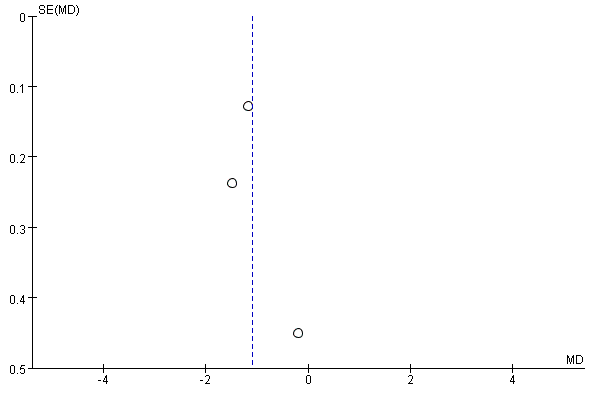
**

**Fig.s6f. Timed Up and Go (TUG).**


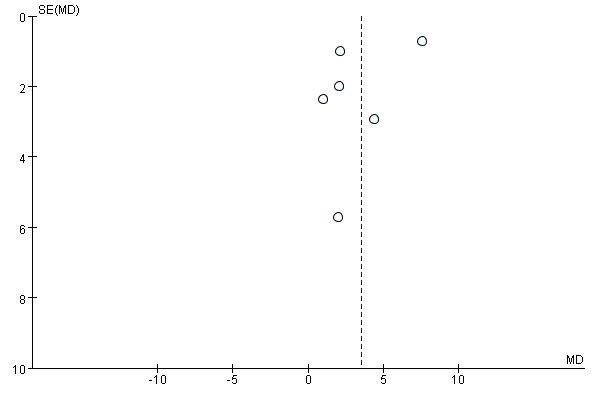


**Fig.s6g. Sit and reach.**


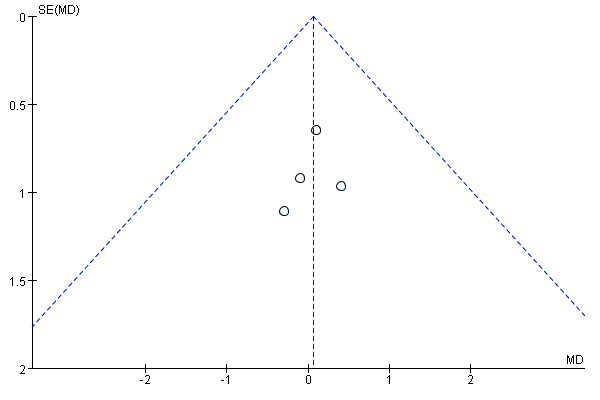


**Fig.s6h. Arm circumference.**


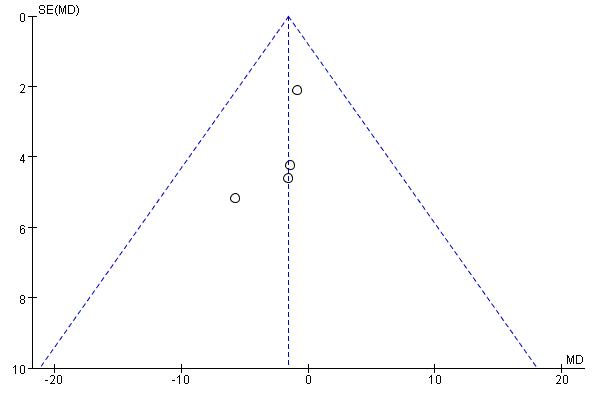


**Fig.s6i.Waist circumference.**


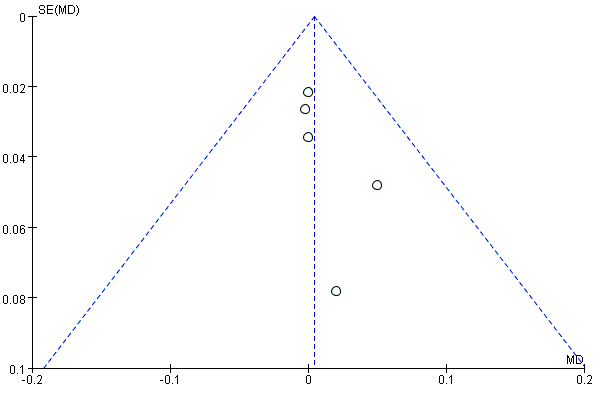


**Fig.s6j. Waist-to-hip ratio.**

**Supplementary table 1. Search strategy**

**Medline searching strategy**  Date 2025-08-01

| Query | Results |
| --- | --- |
| #1 exp Aged/ OR (older adult* or elder* or senior* or geriatric*).tw.  #2 exp Hydrotherapy/ OR exp Swimming/ OR (aquatic exercise* or water exercise* or water-based exercise* or pool exercise* or water aerobics or aquatic therapy or hydro-gymnastics).tw.  #3 exp Body Composition/ OR exp Body Mass Index/ OR exp Muscle Strength/ OR exp Hand Strength/ OR (body composition or body fat or fat mass or lean mass or BMI or muscle strength or grip strength or handgrip strength).tw.  #4 (randomized controlled trial or controlled clinical trial).pt. or (randomized or placebo).ab.  Final Search: #1 AND #2 AND #3 AND #4 | 37 |

**Embase searching strategy** Date 2025-08-01

| Query | Results |
| --- | --- |
| #1 'aged'/exp OR 'elderly'/exp OR 'older adult*':ti,ab OR 'elder*':ti,ab OR 'senior*':ti,ab OR 'geriatric*':ti,ab  #2 'hydrotherapy'/exp OR 'swimming'/exp OR 'aquatic exercise*':ti,ab OR 'water exercise*':ti,ab OR 'water-based exercise*':ti,ab OR 'pool exercise*':ti,ab OR 'water aerobics':ti,ab OR 'aquatic therapy':ti,ab  #3 'body composition'/exp OR 'body mass'/exp OR 'muscle strength'/exp OR 'hand strength'/exp OR 'body composition':ti,ab OR 'fat mass':ti,ab OR 'lean mass':ti,ab OR 'muscle strength':ti,ab OR 'grip strength':ti,ab OR 'BMI':ti,ab  #4 'randomized controlled trial'/exp OR 'randomization'/exp OR 'random*':ti,ab  Final Search: #1 AND #2 AND #3 AND #4 | 69 |

**WOS searching strategy** Date 2025-08-01

| Query | Results |
| --- | --- |
| #1 TS=("older adult*" OR "elder*" OR "senior*" OR "geriatric*" OR "aged")  #2 TS=("aquatic exercise*" OR "water exercise*" OR "water-based exercise*" OR "pool exercise*" OR "hydrotherapy" OR "swimming" OR "water aerobics" OR "aquatic therapy")  #3 TS=("body composition" OR "body fat" OR "fat mass" OR "lean mass" OR "BMI" OR "muscle strength" OR "grip strength" OR "handgrip strength")  #4 TS=("randomized controlled trial" OR "randomised" OR "RCT" OR "clinical trial")  Final Search: #1 AND #2 AND #3 AND #4 | 61 |

**Cochrane Library searching strategy** Date 2025-08-01

| Query | Results |
| --- | --- |
| #1 MeSH descriptor: [Aged] explode all trees  #2 ("older adult*" OR "elder*" OR "senior*" OR "geriatric*"):ti,ab,kw  #3 (#1 OR #2)  #4 MeSH descriptor: [Hydrotherapy] explode all trees  #5 MeSH descriptor: [Swimming] explode all trees  #6 ("aquatic exercise*" OR "water exercise*" OR "pool exercise*" OR "hydrotherapy" OR "swimming" OR "water aerobics"):ti,ab,kw  #7 (#4 OR #5 OR #6)  #8 MeSH descriptor: [Body Composition] explode all trees  #9 MeSH descriptor: [Muscle Strength] explode all trees  #10 ("body composition" OR "BMI" OR "muscle strength" OR "grip strength" OR "fat mass" OR "lean mass"):ti,ab,kw  #11 (#8 OR #9 OR #10)  Final Search: #3 AND #7 AND #11 | 404 |

**Scopus searching strategy** Date 2025-08-01

| Query | Results |
| --- | --- |
| TITLE-ABS-KEY ("older adult*" OR "elder*" OR "senior*" OR "geriatric*" OR aged) AND TITLE-ABS-KEY ("aquatic exercise*" OR "water exercise*" OR "pool exercise*" OR "hydrotherapy" OR "swimming" OR "water aerobics" OR "aquatic therapy") AND TITLE-ABS-KEY ( "body composition" OR BMI OR "fat mass" OR "lean mass" OR "muscle strength" OR "grip strength" OR "handgrip strength") AND TITLE-ABS-KEY ( "randomized" OR "RCT" OR "clinical trial" OR placebo OR "randomly allocated") | 78 |

**Pubmed searching strategy**  Date 2025-08-01

| Query | Results |
| --- | --- |
| #1 "Aged"[Mesh] OR "older adult*"[Title/Abstract] OR "elder*"[Title/Abstract] OR "senior*"[Title/Abstract] OR "geriatric*"[Title/Abstract] OR "aged"[Title/Abstract]  #2 "Hydrotherapy"[Mesh] OR "Swimming"[Mesh] OR "aquatic exercise*"[Title/Abstract] OR "water exercise*"[Title/Abstract] OR "water-based exercise*"[Title/Abstract] OR "pool exercise*"[Title/Abstract] OR "hydrotherapy"[Title/Abstract] OR "swimming"[Title/Abstract] OR "aquatic training"[Title/Abstract] OR "water aerobics"[Title/Abstract] OR "aquatic therapy"[Title/Abstract]  #3 "Body Composition"[Mesh] OR "Body Mass Index"[Mesh] OR "Muscle Strength"[Mesh] OR "Hand Strength"[Mesh] OR "body composition"[Title/Abstract] OR "body fat"[Title/Abstract] OR "fat mass"[Title/Abstract] OR "lean mass"[Title/Abstract] OR "BMI"[Title/Abstract] OR "muscle strength"[Title/Abstract] OR "grip strength"[Title/Abstract]  #4 "Randomized Controlled Trial"[Publication Type] OR "randomized"[Title/Abstract] OR "placebo"[Title/Abstract]  Final Search: #1 AND #2 AND #3 AND #4 | 33 |

**Clinical trial.gov searching strategy** Date 2025-08-01

| Query | Results |
| --- | --- |
| (Aquatic Exercise OR Water Exercise OR Hydrotherapy OR Swimming) AND (Older Adult OR Elderly OR Senior) AND (Strength OR Body Composition OR BMI) | 75 |

**Supplementary Table 2. Results of the meta-regression.**

| Covariate | Coefficient | Standard Error | | 95%CI | |  | Z |  | t |  | *p* |
| --- | --- | --- | --- | --- | --- | --- | --- | --- | --- | --- | --- |
| 30-second chair stand test (30-CST) (n=4) |  |  |  |  |  |  |  |  |  |  |  |
| Treating duration | -0.393 | 2.042 | | -9.177 | 8.392 |  | -0.17 |  | -0.19 |  | 0.865 |
| Treating type | 2.062 | 1.384 | | -3.891 | 8.016 |  | 1.09 |  | 1.49 |  | 0.275 |
|  |  |  |  |  |  |  |  |  |  |  |  |
| Arm curl (n=5) |  |  |  |  |  |  |  |  |  |  |  |
| Treating duration | -0.621 | 3.261 | | -10.998 | 9.757 |  | -0.18 |  | -0.19 |  | 0.861 |
| Treating type | -0.406 | 3.875 | | -12.738 | 11.926 |  | -0.10 |  | -0.10 |  | 0.923 |
|  |  |  |  |  |  |  |  |  |  |  |  |
| Sit and reach (n=6) |  |  |  |  |  |  |  |  |  |  |  |
| Treating duration | -5.357 | 1.092  1.080 | | -8.389 | -2.325 |  | -2.65 |  | -4.91 |  | 0.008 |
| Treating type | 5.343 |  |  | 2.346 | 8.341 |  | 2.65 |  | 4.95 |  | 0.008 |
|  |  |  |  |  |  |  |  |  |  |  |  |
| Triglyceride (n=4) |  |  |  |  |  |  |  |  |  |  |  |
| Treating duration | 0.054 | 0.346  0.383 | | -1.437 | 1.544 |  | 0.14 |  | 0.15 |  | 0.891 |
| Treating type | 0.129 |  |  | -1.517 | 1.776 |  | 0.29 |  | 0.34 |  | 0.768 |
